# Comparative Genomics, Evolutionary and Gene Regulatory Regions Analysis of Casein Gene Family in *Bubalus bubalis*

**DOI:** 10.3389/fgene.2021.662609

**Published:** 2021-03-23

**Authors:** Saif ur Rehman, Tong Feng, Siwen Wu, Xier Luo, An Lei, Basang Luobu, Faiz-ul Hassan, Qingyou Liu

**Affiliations:** ^1^State Key Laboratory for Conservation and Utilization of Subtropical Agro-Bioresources, Guangxi University, Nanning, China; ^2^National Engineering Laboratory for Animal Breeding, Key Laboratory of Animal Genetics, Breeding and Reproduction of the Ministry of Agriculture, College of Animal Science and Technology, China Agricultural University, Beijing, China; ^3^Shannan Animal Husbandry and Veterinary Terminus, Xizang, China; ^4^Faculty of Animal Husbandry, Institute of Animal and Dairy Sciences, University of Agriculture, Faisalabad, Pakistan

**Keywords:** buffalo breeds, caseins, evolution, regulatory regions, milk yield

## Abstract

Buffalo is a luxurious genetic resource with multiple utilities (as a dairy, draft, and meat animal) and economic significance in the tropical and subtropical regions of the globe. The excellent potential to survive and perform on marginal resources makes buffalo an important source for nutritious products, particularly milk and meat. This study was aimed to investigate the evolutionary relationship, physiochemical properties, and comparative genomic analysis of the casein gene family (*CSN1S1, CSN2, CSN1S2*, and *CSN3*) in river and swamp buffalo. Phylogenetic, gene structure, motif, and conserved domain analysis revealed the evolutionarily conserved nature of the casein genes in buffalo and other closely related species. Results indicated that casein proteins were unstable, hydrophilic, and thermostable, although αs1-CN, β-CN, and κ-CN exhibited acidic properties except for αs2-CN, which behaved slightly basic. Comparative analysis of amino acid sequences revealed greater variation in the river buffalo breeds than the swamp buffalo indicating the possible role of these variations in the regulation of milk traits in buffalo. Furthermore, we identified lower transcription activators STATs and higher repressor site YY1 distribution in swamp buffalo, revealing its association with lower expression of casein genes that might subsequently affect milk production. The role of the main motifs in controlling the expression of casein genes necessitates the need for functional studies to evaluate the effect of these elements on the regulation of casein gene function in buffalo.

## Introduction

Buffalo is a luxurious genetic resource with multiple utilities (as a dairy, draft, and meat animal) and economic significance in the tropical and subtropical regions of the globe (Rehman et al., 2019, 2020; Luo et al., 2020). The domesticated buffalo is grouped into river buffalo with karyotype 2n = 50 primarily present in southwestern Asia, India, south Mediterranean Europe, and Egypt and swamp buffalo with 2n = 48 distributed across Southeast Asia, southern and southeast China, where the swamp buffalo is used as draft power in the rice paddy fields while the river buffalo is mainly reared for milk production (Moioli et al., 2001; Fan et al., 2020; Luo et al., 2020). The excellent potential to survive and perform on marginal resources under harsh environmental conditions makes buffalo an important source for nutritious products, particularly milk and meat. Buffalo contributes about 13% of global milk production where the river buffalo produces 2,000 kg milk per year and swamp buffalo annual production is 500–600 kg (Basilicata et al., 2018; Fan et al., 2020; Lu et al., 2020). Moreover, the physio-chemical characteristics of buffalo milk are different from cow milk, and buffalo milk is relished due to its peculiar taste and higher butterfat content (Li et al., 2020).

Buffalo milk contains higher protein, fat, and total solid contents relative to dairy cow milk (Ahmad et al., 2013). The milk proteins are broadly categorized into whey (serum) protein and casein protein families based on their physio-chemical properties. Casein (CN) is the major milk protein, contributing 80% of the whole milk proteins including α-s1-CN, α-s2-CN, β-CN, and k-CN. Each CN protein has its unique amino acid configuration, genetic and functional properties (Fan et al., 2020). Milk CNs are physiologically important as they provide food to the newborn and are associated with milk processing properties and lactation behaviors of dairy animals (Nilsen et al., 2009).

Notably, the CN protein is characterized into calcium-sensitive αS1, αS2, and β caseins, in young one sustenance bone growth through providing calcium, and phosphorus enriched stable micelles, and the Ca-insensitive κ-casein (Pauciullo and Erhardt, 2015). So far, in mammals, caseins are the main constituent of milk proteins. The casein proteins coding genes CSN1S1 (αs1-casein), CSN1S2 (αs2-casein), CSN2 (β-casein), and CSN3 (κ-casein), have been mapped in the 250-350kb genomic DNA cluster on chromosome 6 in sheep, goat, and cattle (Rijnkels, 2002).

Casein is considered a powerful molecular model for evolutionary research (Kawasaki et al., 2011). It is also a useful tool to better understand the genetic architecture of less-studied species, phylogenetic relationships among mammalian species, and domestic animals, particularly the buffalo breeds (rive and swamp). From a physiological standpoint, there is a difference in milk yield and composition traits, including protein, fat, and solid contents among different species or breeds, suggesting the potential role of gene regulatory regions in these breeds. Exploring the genetic architecture and evolutionary processes is imperative to understand the regulatory mechanisms of the casein gene family in the buffalo. This study aims to investigate the evolutionary relationship, physiochemical properties, comparative genomics, and gene regulatory regions analysis of the casein gene family in river and swamp buffalo.

## Materials and Methods

The sequences of different casein genes (*CSN1S1, CSN2, CSN1S2*, and *CSN3*) of *Bos taurus* were retrieved from NCBI^1^ and used as queries for the identification of casein genes from the buffalo genome. The buffalo (river and swamp) whole-genome sequences were downloaded from the Bigdata center and NCBI^1,2^. The *Bos taurus* casein protein sequences (XP_005208084.1, XP_024848786.1, XP_010804480.2, and XP_024848756.1) were used in BLAST search with an E value less or equal to 1.0 × e^–5^ with all default parameters, to retrieve non-redundant protein sequences of the buffalo. To avoid ambiguity, the redundancy of the sequences was checked. The chromosomal locations of casein genes were obtained from buffalo genome resources through the GFF file of annotated buffalo genome with corresponding gene positions in the MCScanX program as reported earlier (Wang et al., 2012).

The Maximum Likelihood method based on the JTT matrix model was used to infer the evolutionary history of representative species (Jones et al., 1992). The accessions number of amino acid sequences used to construct the phylogenetic tree and holology of the representative speices sequence are given in Supplementary Table S1. The likelihood phylogram of 44 amino acid sequences with the highest log (−1641.52) was downloaded and the percentage of trees in which the associated taxa clustered together presented next to the branches. A bootstrap value of 3,000 replicates was used and the percentage of resampling was visualized on the node of the phylogram. All the missing and gaped positions were eliminated and MEGA7 was used to conduct the evolutionary analyses (Kumar et al., 2016).

Moreover, the genomic and coding sequence data of casein genes from buffalo and cattle were submitted to Gene Structure Display Server 2.0^3^, for gene structure analysis and visualization of untranslated regions and exon-intron structure (Hu et al., 2015). Additionally, 10 MEME (Multiple EM for Motif Elicitation) conserved motifs of caseins were explored using the MEME Suite^4^ (Bailey et al., 2006). The NCBI conserved domain (CDD) database was used to confirm the conserved domains^5^.

ProtParam tool was used to illustrate the physio-chemical properties of buffalo casein proteins including the isoelectric point (pI), grand average of hydropathicity (GRAVY), molecular weight (MW), number of amino acids, instability index (II), and aliphatic index (AI) (Gasteiger et al., 2003). Multiple sequence alignment of casein protein sequences was performed in Multiple Align Show to visualize the sequence variations and indels^6^.

The genomic sequences of casein genes of Mediterranean and swamp buffalo were subjected to the Promoter 2.0 Prediction Server^7^ to detect potential signals for putative transcription binding factor. The site with a score >1.0 was presumed as a high likelihood predicted site and the putative transcription binding factor site sequence was searched in the 100bp upstream regions from the high likelihood predicted site (Knudsen, 1999). Further, the genomic sequences were analyzed in TFBIND software^8^ by using the transcription factor database TRANSFAC R.3.4 weight matrix to find the transcription factor binding sites (Tsunoda and Takagi, 1999). According described previously, four potential transcription factor binding sites (GATA, TATA, STAT, and OCT1) (Hennighausen and Robinson, 1998; Robinson et al., 1998; Rosen et al., 1999; Wheeler et al., 2001; Wyszomierski and Rosen, 2001; Yamashita et al., 2001; Chughtai et al., 2002; Pauciullo et al., 2019) and one repressor site (YY1) (Helman et al., 1998; Tomic et al., 1999) in casein genes of Mediterranean and swamp buffalo in 100bp upstream regions of the potential signal site were calculated (Wyszomierski and Rosen, 2001). The significant difference for the distribution of putative transcription factor binding and repressor sites in Mediterranean and swamp buffalo was statistically evaluated by using a *t*-test with a *P-*value of < *0.05* as statistical significance. Moreover, the potential nuclear hormone receptor sites in the genome of Mediterranean buffalo were detected by using the NHR scan^9^.

## Results

**FIGURE 1:**
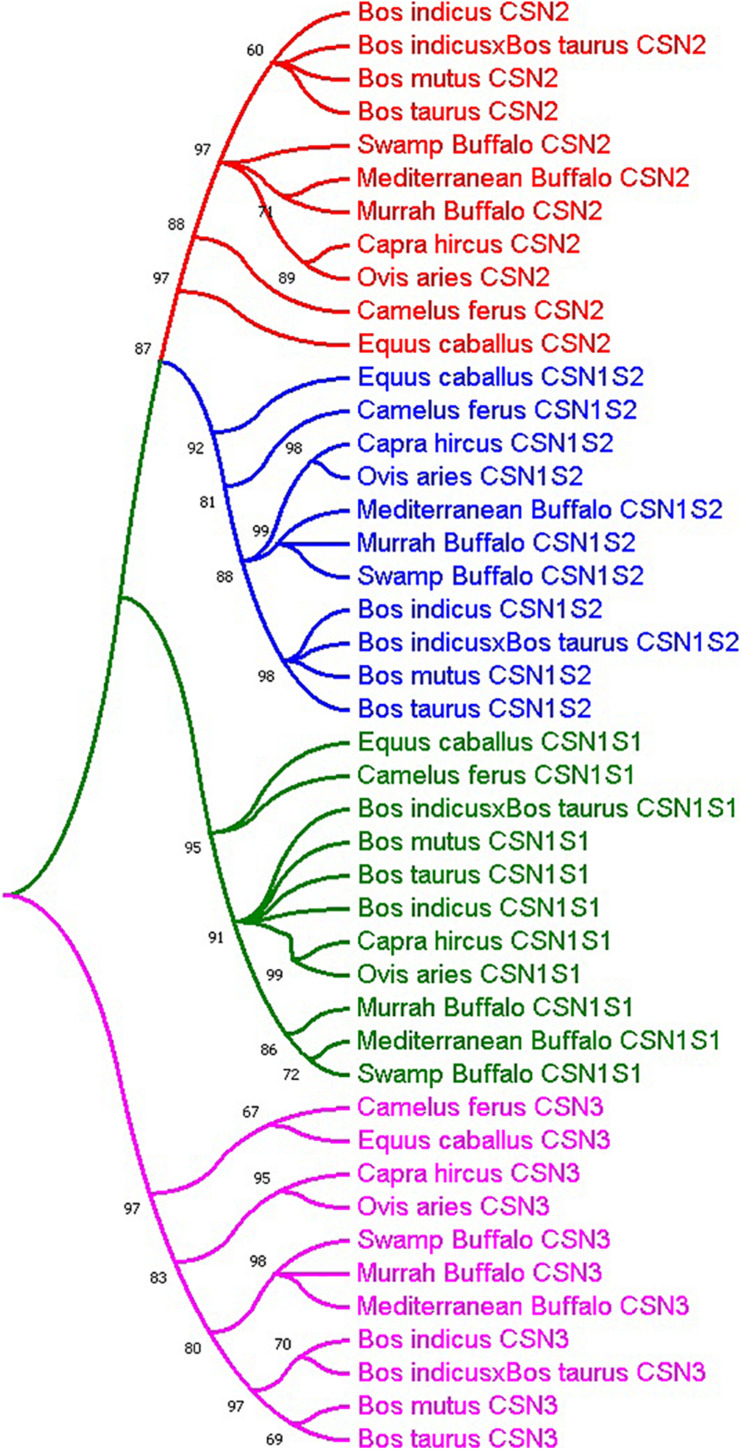
Molecular phylogenetic analysis of casein gene family (green; *CSN1S1*, blue; *CSN1S2*, red; *CSN2* and fuchsia; *CSN3*) in representative species.

The molecular phylogenetic analysis of representative bovine species revealed that all the casein gene sequences were clustered into four groups; *CSN1S1*, *CSN2*, *CSN1S2*, and *CSN3* (Figure 1). Additionally, overall phylogenetic relationships revealed that *Bubalus bubalis CSN* gene family is more closely related to *Bos mutus, Bos taurus*, and *Bos indicus* sharing higher sequence homology about 93, 91, and 90%, respectively, as compared to the *Capra hircus, Ovis aries* and hybrid cattle with 86, 84, and 74% similarity respectively. Moreover, distantly related species included *Camelus ferus* and *Equus caballus* with 55 and 50% resemblance, respectively (Supplementary Table S2).

**FIGURE 2:**
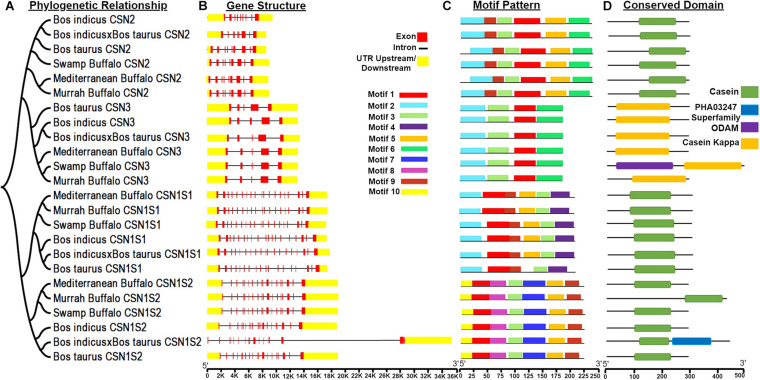
Phylogenetic relationships, gene structure, motif patterns, and conserved domain regions of the casein proteins gene family. **(A)** Phylogenetic relationship of 24 amino acid sequences of casein proteins. **(B)** Gene structure of casein. **(C)** Motif pattern. **(D)** Conserved domain regions of the casein proteins gene family. Ten putative motifs are indicated in different colored boxes. For details of motifs refer to Table 1.

Furthermore, to perform the structural characterization of the *CSN* gene family in different species, analysis of gene organization, motifs pattern, and the conserved domains were carried out considering their phylogenetic relationships (Figure 2). In casein genes, 10 MEME conserved motifs were identified (Figure 2C). Motif 3 corresponding to 21 amino acid was annotated as kappa casein (K-CN) domain while motif 4, 5, and 6 were annotated as casein domain after the Pfams search (Table 1). The CDD BLAST was used to confirm the identified conserved domains (Figure 2D). Additionally, the ODAM and PHA03247 superfamily domain has also been dredged up in *CSN* genes (Figure 2D). Besides, the upstream and downstream untranslated regions (UTRs) and intron structure considerably varied, structural analysis of the gene indicated that buffalo *CSN* genes in the same group possess a corresponding number of introns and exons (Figure 2B). However, different *CSN* gene groups exhibited a variable pattern of introns and exons (Figure 2B).

**TABLE 1:** Ten differentially conserved motifs detected in casein protein (*CSN1S1, CSN1S2, CSN2*, and *CSN3*) gene family.

Motif	Protein sequence	Length	Pfam domain
MEME-1	NTLPENISSAEETDVAREPYKQLEAMAISPSKEALAT	37	–
MEME-2	MKLLILTCLVALALARPLEELKVQGEPQEVLNENEERFFVA	41	–
MEME-3	BKYQQKELALINNQYLAYPYY	21	K-CN
MEME-4	FRQFYQLDAYPSGAWYYVPLGTQYTDAPSFSDIPNPIGSENSGKTTMPLW	50	CN
MEME-5	VEVFTEKTKLTEEDVERLNLLKKJSQSYMHFPK	33	CN
MEME-6	IPSINKILPVEPKAVPYPZADEPIVAFLEYSEEVJGPVPEP	41	CN
MEME-7	QYLYQGPIVLNPWDQVKRNAVPITPTLNR	29	–
MEME-8	TFCKEVVRNANEEEYSIGSSSEESAEVAT	29	–
MEME-9	NKEVEKFQKEEKPST	15	–
MEME-10	MKFFIFTCLLAVALA	15	–

**K-CN; kappa casein, CN; Casein.**

Physiochemical properties of the *CSN* gene family in *Bubalus bubalis* was determined in terms of their distribution on the chromosome, exon count, molecular weight (Da), number of the amino acids (A.A) in each peptide, aliphatic index (AI), isoelectric point (pI), instability index (II) and Grand Average of hydropathicity Index (GRAVY) (Table 2). All the *CSN* genes were found on chromosome 7 in the region between ∼250 kb that harbors a variable number of exons and inconsistent length of the gene with amino acid residues (Table 2). The molecular weight of CN proteins ranged from 21 to 29 kDa. The CN peptides in buffalo were observed as unstable but thermostable proteins as the aliphatic index for all caseins had values > 65. Further, the pI values revealed that all CN proteins αs1-CN, β-CN, and κ-CN were acidic peptides except αs2-CN which behaved slightly basic in nature (Table 2). Lower values of GRAVY indicate the hydrophilic nature of buffalo CN proteins (Table 2).

**TABLE 2:** Physiochemical properties of the casein gene family in *Bubalus bubalis* (Mediterranean breed).

Buffalo breed	Gene	Chromosome	Exon count	MW (Da)	A.A	pI	AI	II	GRAVY
Italian	CSN1S1	7	19	23451.87	206	4.89	90.87	59.32	−0.332
Italian	CSN1S2	7	18	25081.53	213	7.66	73.66	45.54	−0.699
Italian	CSN2	7	9	29110.29	259	6.31	100.04	92.21	−0.124
Italian	CSN3	7	5	21409.62	190	6.83	86.21	49.60	−0.232

**MW, Molecular Weight in Daltons; A.A, number of amino acids; pI, Isoelectric point; AI, Aliphatic Index; II, Instability Index; and GRAVY, Grand Average of hydropathicity Index.**

**FIGURE 3:**
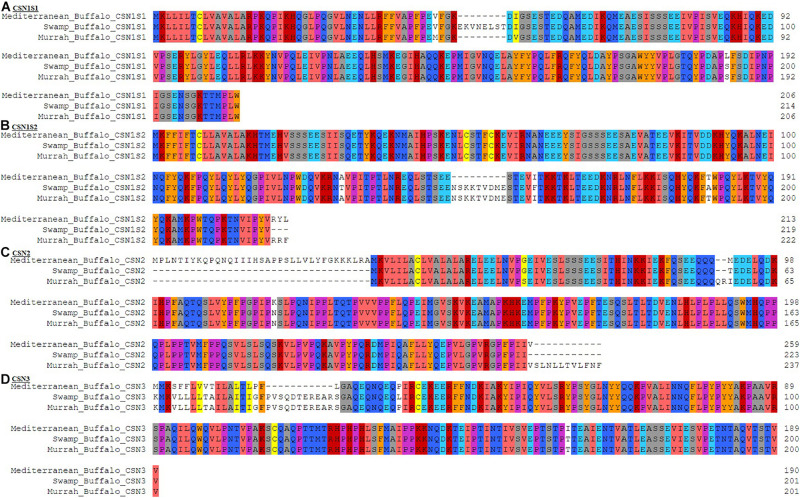
Comparative amino acid analysis of casein gene family in Mediterranean, Swamp, and Murrah buffalo breeds. **(A)** *CSN1S1* gene, **(B)** *CSN1S2* gene, **(C)** *CSN2* gene, **(D)** *CSN3* gene.

Comparative amino acid analysis of buffalo breeds revealed 7 indels in *CSN* genes including a single indel in both *CSN1S1* and *CSN3* while two indels in *CSN1S2* and 3 in *CSN2*. The *CSN1S1* gene has an indel of 8 amino acids at position 50 > 57 whereas single amino acid change V46 > M in Murrah and S193 > L in Mediterranean buffalo was also observed (Figure 3A). Two indels of variable length were in CSN1S2, where 9 amino acid indel is positioned at 149 > 157, presumably is due to an alternative splicing of exon 13, and the second indel toward the terminal end of the peptide with a length of three amino acids 220 > 222. In swamp buffalo, three amino acid variations A131 > T, I162 > F, and T190 > A were also detected in *CSN1S2* (Figure 3B). Furthermore, in *CSN2* two prominent indels toward terminal ends with a length of 35 amino acids (5′end) at 1 > 35 and 12 amino acids (3′end) at 261 > 272, and a short indel of 2 amino acids 91 > 92 was observed. A single amino acid modification was observed in the Mediterranean buffalo (N120 > K) but much variable amino acid in three buffalo breeds was observed at 93 M > T > I (Figure 3C). Moreover, a highly variable region toward the 5′ end in *CSN3* was perceived with an indel of 11 amino acids 19 > 29. All single amino acid differences were marked in Mediterranean buffalo except P40 > L which is observed in swamp buffalo (Figure 3D).

**FIGURE 4:**
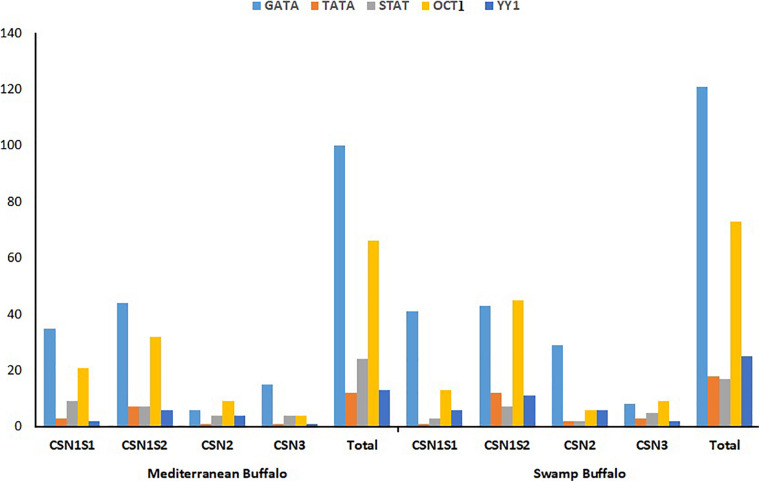
Comparative distribution of putative transcription binding site (GATA, TATA, STAT, and OCT1) and repressor site (YY1) in the genomic sequences of Mediterranean and Swamp buffalo casein gene family.

The genome sequences of Mediterranean and swamp buffalo *CSN* gene family was scanned to find out putative transcription factors binding sites by selecting previously reported four potential transcription sites (GATA, TATA, STAT, and OCT1), and one repressor binding site (YY1) (Supplementary Tables S3, S4). Both Mediterranean and swamp buffalo shared approximately an equal number of respective transcription sites except the repressor site YY1 that was highly distributed (*P* < 0.05) in the swamp buffalo as compared to the Mediterranean buffalo (Figure 4 and Supplementary Table S5). The distribution of GATA in the Mediterranean was 35, 6, 44, and 15 correspondings to *CSN1S1, CSN2, CSN1S2*, and *CSN3*, respectively, while swamp buffalo had 41, 29, 43, and 8, respectively (Figure 4 and Supplementary Table S5). Furthermore, TATA site distribution in Mediterranean buffalo was 3, 1, 7, and 1 in *CSN1S1, CSN2, CSN1S2*, and *CSN3*, respectively but in swamp buffalo, it was 1, 2, 12, and 3, respectively (Figure 4 and Supplementary Table S5). A considerable difference (*P* > 0.05) was observed in the STAT site’s distribution in *CSN1S1* (9 vs. 3), *CSN2* (4 vs. 2), *CSN1S2* (7 vs. 7), and *CSN3* (4 vs. 5) of Mediterranean and swamp buffalo (Figure 4 and Supplementary Table S5). The distribution of OCT1 transcription sites varied across the *CSN1S1* (21 vs. 13), *CSN2* (9 vs. 6), *CSN1S2* (32 vs. 45), and *CSN3* (4 vs. 9) of Mediterranean and swamp buffalo (Figure 4 and Supplementary Table S5).

**FIGURE 5:**
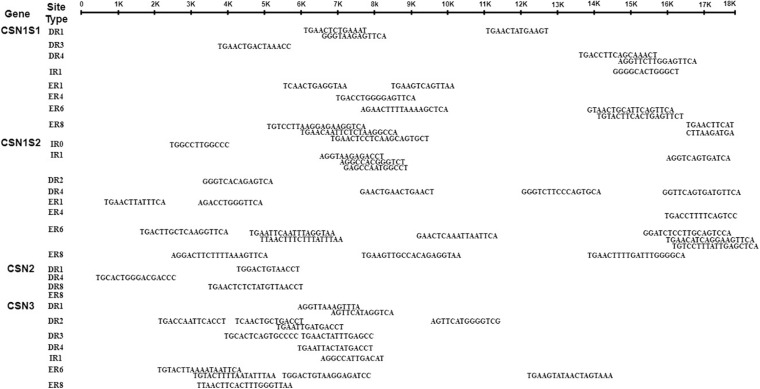
Nuclear hormone receptor sites patterns in the casein gene family of *Bubalus bubalis*.

The pattern of nuclear hormone receptors (NHRs) sites in the *CSN* gene family of *Bubalus bubalis* was explored using genome sequence data of Mediterranean buffalo. A total of 58 NHRs sites were observed in the buffalo *CSN* gene family that was mostly distributed toward 5′end (Figure 5 and Supplementary Table S6). Moreover, the number of NHRs identified in *CSN1S1, CSN1S2, CSN2*, and *CSN3* were 17, 22, 4, and 15, respectively (Figure 5). A total of 7 inverted repeats (IR) were observed in different *CSN* genes that are primarily used as the hormonal response element (HRE) important for steroid receptors. Single IR in each of *CSN1S1* and *CSN3*, while 5 IR were observed in *CSN1S2* whereas, *CSN2* harbored no IR (Figure 5 and Supplementary Table S6). In total 22 direct repeats (DR) and 29 everted repeats (ER) were found in the buffalo *CSN* genes which are prominently used by type II receptors (RXR) and some type III receptors (orphan receptors) can also able to use DR. The number of DR distributed in *CSN1S1, CSN1S2, CSN2*, and *CSN3* was 6, 4, 3, and 9, and ER was 10, 13, 1, and 5, respectively (Figure 5 and Supplementary Table S6). All these HRE were detected close to the putative transcription binding sites (Supplementary Tables S3, S4, S6).

## Discussion

The advances in genome sequencing technology particularly next-generation sequencing has led to the availability of sequenced genomes for different animal species that opens up new ways to understand genomic architecture at the molecular level (Luo et al., 2020). Comparative genomics provides an opportunity for discovering novel genes and their functional components (Wei et al., 2002; Rijnkels et al., 2003). Exploring the genetics and evolutionary processes is required to understand the regulatory mechanisms of different physiological important genes like the *CSN* gene family in mammals. Buffalo possesses significant economic attributes owing to its high milk protein contents which are imperative for the production of commercial dairy products like cheese. The milk proteins and related coding genes have been ubiquitously studied due to their extensive distribution in all mammalian species, as an enriched nutrient source for neonates. Caseins (αs1, αs2, β, and κ) are the primary components of milk protein content in dairy animals. All the mammalian *CSN* genes are rapidly evolving genes and are mainly classified into four types including *CSN1S1, CSN2*, *CSN1S2*, and *CSN3* (Madende and Osthoff, 2019). The results of our molecular phylogenetic analysis of the *CSN* gene family are in consensus as all the representative species were clustered into four taxa. The buffalo species were grouped with cattle, *Capra hircus*, and *Ovis aries* sharing higher sequence homology with cattle breeds (Figure 1).

The amino acid sequence of protein data can impersonate a better prototype of biologically substantial conserved evolutionary motifs. For protein structural and functional analyses, these conserved regions are vital and can be traced by Multiple sequence alignment (Neuwald, 2016). In reference to the aligned sequence of the *CSN* gene, high variation has been reported in all the *CSN* genes. Even though closely related species represent increased sequence similarity with conserved and non-conserved genomic regions (Madende and Osthoff, 2019). In the present study, sequence analysis of CN protein revealed 10 conserved motifs in buffalo, and cattle using the MEME tool. Apart from the sequence variations in the *CSN* gene, further differences and divergence were observed because of different incidents including exon skipping (Martin and Orgogozo, 2013). Besides, the upstream and downstream UTRs and introns structure considerably varied, structural analysis of the gene indicated that buffalo *CSN* genes in the same group have a consistent number of exons and introns but variable patterns of exons and introns have also been observed. The variability of UTRs and intronic regions is mostly because of the absence or presence of retroposonic elements. In fact, these ruminants-specific retrotransposons insertions are often polymorphic (absent or present) at orthologous loci and they are highly informative genetic markers that can be considered a powerful phylogenetic tool for clustering studies, animal evolutionary history, population structure, and demography. In general, these elements are known to affect the genome in many other different ways: contributing to the genome size increase, genomic instability, exonization, epigenetic regulation, RNA editing, and so on (Cosenza et al., 2009; Giambra et al., 2010).

All these caseins are encoded by autosomal genes *CSN1S1, CSN2*, *CSN1S2*, and *CSN3*, respectively in closely linked DNA clusters (Pauciullo et al., 2019). The genomic cluster of the casein gene spans between 250 and 350 kb in different mammalian species (Ryskaliyeva, 2018), and in buffalo entire *CSN* gene covers a region of 250kb. This was hypothesized that the exon duplications events in the ancestral gene result in casein gene evolution (Jones et al., 1985). For instance, donkeys, horses, rabbits, and rodents possess an extra copy of αs2-casein indicating the event of recent paralogous gene duplication (Stewart et al., 1987; Ginger et al., 1999). While no evidence for the paralogous gene duplication in buffalo was practically observed that confirms the previous findings of phylogenetic data, which demonstrated Artiodactyla gene loss, whereas gain of an extra copy of the gene in other species was somewhat attained by differential exon usage (Rijnkels, 2002). Caseins are intrinsically disordered proteins (IDPs) related groups of proteins, manifested in milk as roughly spherical, amorphous, polydisperse particles, classically encompassing protein chains, and calcium phosphate nanoclusters. These particles are termed as casein micelles (Cosenza et al., 2010). Caseins have flexible open conformation with an abundance of poly-L-proline II secondary structures and cannot be considered as hydrophobic proteins (Carver and Holt, 2019). Similarly, lower values of GRAVY represent the hydrophilic nature of buffalo CN proteins.

Moreover, short phosphorylated sequences and flexible conformation remarkably increase the casein’s ability to keep calcium phosphate nanoclusters and develop a dense shell of peptide around the calcium phosphate to form a thermodynamically stable core-shell complex, even at quite higher phosphate and calcium concentrations (Carver and Holt, 2019). In the present study, the aliphatic index showed that all CN proteins have values >65 so perused as thermostable. The casein micelles formation is essential for the effective transportation of phosphate and calcium via milk from the mother to the neonate (Holt et al., 2013). Thus, a readily digestible calcium-enriched diet in the form of casein micelle is available for the neonate. Caseins as IDPs play an important role in mammary gland protection against pathological calcification, amyloid formation, and other dysfunctional processes that can minimize the reproductive success of the mother (Carver and Holt, 2019). Our findings illustrated all the CN peptides in buffalo were determined as unstable protein and the pI revealed all casein proteins α s1-, β-, and κ-CN were determined as acidic peptides except αs2 which behaved slightly basic nature.

In recent years, the polymorphisms of milk proteins have aroused great research interest because of the genotypes of milk proteins may be related to milk composition and milk yield of dairy mammals (Nilsen et al., 2009). The amino acid changes possibly have a functional effect on the buffalo caseins (Fan et al., 2020). Comparative amino acid sequence analysis revealed that CN protein harbor higher amino acid variations in river buffalo (Mediterranean and Murrah) as compared to the swamp buffalo. The results of the present study are in line with previous studies (Masina et al., 2007; Azevedo et al., 2008; Massella et al., 2017; Rangel et al., 2017; Miluchova et al., 2018; Fan et al., 2020; de Oliveira et al., 2021) which reported the potential association of genetic variants in *CSN* genes with lactation performance, milk composition, and attributes of milk products. Thus, casein gene-based markers are important candidates for the selective breeding of buffalo to improve the quantity and quality of milk (de Oliveira et al., 2021). Moreover, further insights are required to ubiquitously apply these candidate markers to other mammals due to genetic variability and locus distribution (Sulimova et al., 2007; Cosenza et al., 2015).

Nevertheless, understanding the molecular basis for the regulation of *CSN* gene expression is very crucial for improving milk production (Debeljak et al., 2005). Sequence analysis of promotor region of *CSN* genes has shown various transcription factor binding sites including transcription initiation sites such as STAT5, NF1 and GR, C/EBP (Hennighausen and Robinson, 1998; Robinson et al., 1998; Rosen et al., 1999; Wheeler et al., 2001; Wyszomierski and Rosen, 2001; Yamashita et al., 2001; Chughtai et al., 2002) and potential repressors sites such as YY1, CIS3, SOCS-1, and SOCS-3 (Helman et al., 1998; Tomic et al., 1999). The identification of critical regulatory regions responsible for the expression of the *CSN* genes provides valuable information for the selection of markers in dairy mammals especially the buffaloes. So, in both Mediterranean and swamp buffalo, we selected four potential transcription sites (GATA, TATA, STAT, and OCT1) and one repressor binding site (YY1), for comparative genomic analysis (Figure 4). OCT1 affects the factors of acute myeloid leukemia (AML), forming a complex that reduces its inhibitory role in DNA binding and promotes the expression of the casein gene (Inman et al., 2005).

**FIGURE 6:**
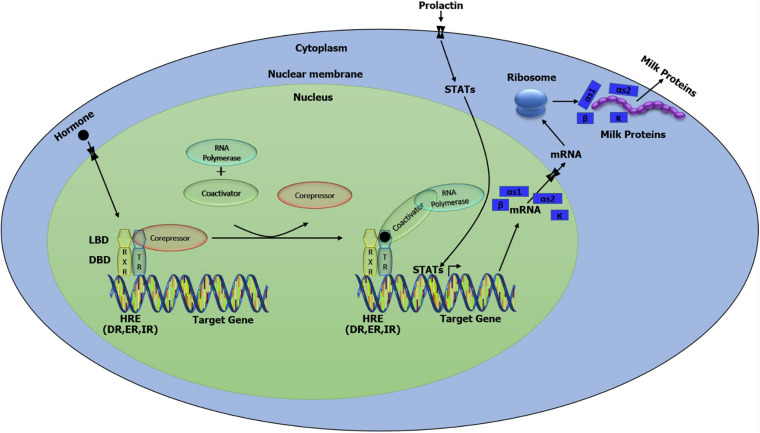
Putative model of the network involved in the regulation and biosynthesis of casein protein in buffalo mammary tissue.

Various lactogenic hormones like prolactin, insulin, hydrocortisone, and some growth factors such as insulin-like growth factor 1 (*IGF-1*) and epidermal growth factor (*EGF*) are crucial for mammary gland activation and eventually the milk proteins gene expression regulation (Hennighausen and Robinson, 1998; Tsunoda and Takagi, 1999). Therefore, we further analyzed the distribution of HRE including DR, ER, and IR in the genome of the buffalo. All these HRE were detected close to the putative transcription binding sites. Therefore, the combined action of the transcription factor and HRE can mediate the activation of caseins (Figure 6). STAT5 is the principal transcription factor in milk protein gene expression that could be activated by the action of growth hormone (*GH*) and prolactin (*PR*) via the STAT/JAK2 signaling pathway or Src-kinase/STAT signaling pathway through the *EGF* action (Gallego et al., 2001). Dimerization and phosphorylation activate the STAT5 and translocate it to the nucleus where STAT5 dimers bind with the DNA and induce transcription (Figure 6; Gallego et al., 2001).

**FIGURE 7:**
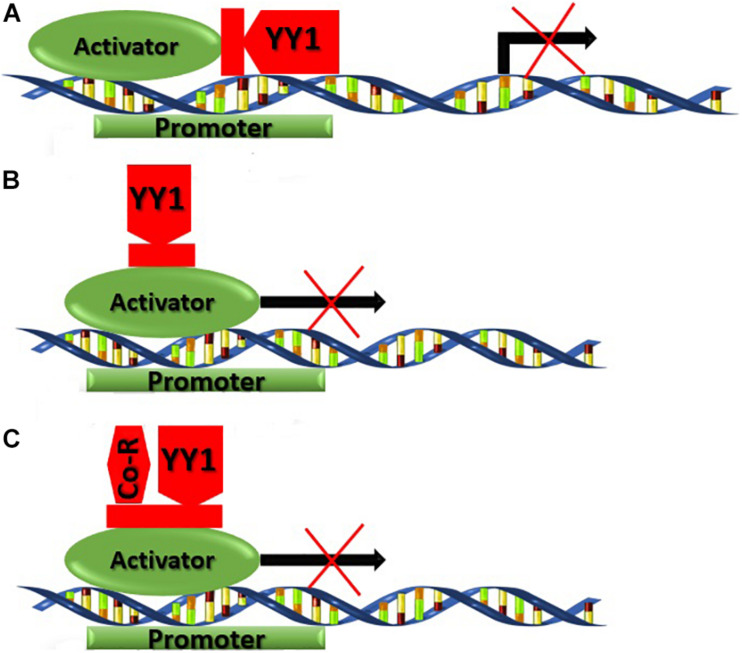
Different transcriptional suppression mechanisms used by YY1.

Multiple mechanisms are being used by YY1 for transcriptional suppression. Mostly YY1 competes with activator factors and overlaps the binding site ultimately repressing the gene transcription. In mammary epithelial cells, YY1 competes with a β-CN activating promoter also known as mammary gland factor (MGF), fallouts in transcription repression (Figure 7A). Moreover, the c-fos promoter possesses two extra YY1 sites between the TATA box and calcium or cyclic AMP response element (CRE) in addition to YY1 overlapping sites (Gordon et al., 2006). The YY1 binding remotely caused direct suppression of the upstream CRE promoter. YY1 can repress the c-fos promoter in a site-dependent or independent manner, including the interaction of zinc finger patterns or binding with cAMP response element-binding (CREB) at the basic leucine zipper region (bZIP) in YY1 (Figure 7B). Most likely, the YY1 and CREB interact in the nucleus and inhibit transcription (Gordon et al., 2006). The YY1 can recruit corepressors that directly induce transcriptional repression or facilitate chromatin condensing to assist further YY1 mediated repression. The repression activity of YY1 is generally because of its glycine-rich and zinc-finger regions. Simultaneous deletions in each individual or both regions reduce the GAL4-YY1 fusion proteins deficient for transcriptional repression (Figure 7C). Thus, cofactors interactions are often required with repression domains of YY1 to facilitate repression like mRPD3 (Yang et al., 1996) or Smad family members (Kurisaki et al., 2003). A considerably higher ratio of STATs distribution and lower number of repressor binding site YY1 was observed in Mediterranean buffalo as compared to swamp buffalo. This envisages that lower STATs and higher YY1 site distribution in swamp buffalo might lead to a lower expression of *CSN* gene subsequently leading to poor milk yield in swamp buffalo.

Our study provides inclusive insights into the regulation of the casein gene family revealing a plausible association of STATs and YYI distribution with a poor milk production potential of swamp buffalo. Moreover, we report striking findings regarding genetic variations in transcription activators and repressor elements from evolutionary standpoint. Further investigations are required to confirm these findings to elucidate the putative role of STATs and repressor sites in the regulation of *CSN* gene expression and their potential utility for the genomic selection of buffaloes for effective utilization and enhanced production.

## Conclusion

The present study provides a comprehensive insight into the molecular structure and function of the casein gene family in buffalo. Phylogenetic, gene structure, motif, and conserved domain analysis elucidated the evolutionary conserved nature of the casein gene in buffalo and closely related species. Buffalo casein proteins were observed as unstable, hydrophilic, and thermostable. The α s1-, β-, and κ-CN behaved as acidic peptides except for αs2, which was slightly basic. Comparative genomic analysis revealed higher amino acid variations in the river buffalo (Mediterranean and Murrah breeds) than swamp buffalo, revealing that these variations may influence milk production traits in buffalo. Moreover, for the first time, our findings indicate lower STATs and higher YY1 site distribution in swamp buffalo as a plausible reason for the comparatively lower expression of casein genes that ultimately affect milk production.

## Data Availability Statement

The datasets presented in this study can be found in online repositories. The names of the repository/repositories and accession number(s) can be found in the article/Supplementary Material.

## Author Contributions

FH and QL: conceptualization. SR, TF, and QL: resources. FH, BL, TF, and XL: data curation. SR, TF, and XL: methodology and software. QL and AL: supervision. SR: writing—original draft preparation. FH, XL, SW, TF, AL, BL, and QL: writing—review and editing. All authors have read and agreed to the published version of the manuscript.

## Conflict of Interest

The authors declare that the research was conducted in the absence of any commercial or financial relationships that could be construed as a potential conflict of interest.

## Footnotes

1 https://www.ncbi.nlm.nih.gov/

2 https://bigd.big.ac.cn

3 http://gsds.gao-lab.org/

4 http://meme-suite.org/tools/meme

5 https://www.ncbi.nlm.nih.gov/Structure/cdd/wrpsb.cgi

6 https://www.bioinformatics.org/sms/multi_align.html

7 http://www.cbs.dtu.dk/services/Promoter/

8 http://tfbind.hgc.jp/

9 http://www.cisreg.ca/cgi-bin/NHR-scan/nhr_scan.cgi
