# Supplementary material for: Comparative Genomics, Evolutionary and Gene Regulatory Regions Analysis of Casein Gene Family in Bubalus bubalis
Source: Front Genet. 2021 Mar 23;12:662609. doi: 10.3389/fgene.2021.662609 (PMC8021914; doi:10.3389/fgene.2021.662609)
Supplement: Supplementary file 1 [file Data_Sheet_1.ZIP › Supplementary file-Revised.docx]

**Table S1.** The accession number of amino acid sequences used to find the percentage of sequence homology in representative species and phylogenetic tree

| **Specie** | **CSN1S1** | **CSN1S2** | **CSN2** | **CSN3** |
| --- | --- | --- | --- | --- |
| **Bos indicus** | XP_019818428.1 | [XP_019818430.1](https://www.ncbi.nlm.nih.gov/protein/XP_019818430.1) | XP_019818429.1 | XP_019818432.1 |
| **Bos indicus X Bos taurus** | XP_027400470.1 | [XP_027401112.1](https://www.ncbi.nlm.nih.gov/protein/XP_027401112.1) | XP_027400471.1 | XP_027400473.1 |
| **Bos mutus** | XP_005902100.1 | XP_014335716.1 | XP_005902099.2 | [XP_005897104.1](https://www.ncbi.nlm.nih.gov/protein/XP_005897104.1) |
| **Bos taurus** | [XP_005208084.1](https://www.ncbi.nlm.nih.gov/protein/XP_005208084.1) | [XP_024848786.1](https://www.ncbi.nlm.nih.gov/protein/XP_024848786.1) | [XP_010804480.2](https://www.ncbi.nlm.nih.gov/protein/XP_010804480.2) | [XP_024848756.1](https://www.ncbi.nlm.nih.gov/protein/XP_024848756.1) |
| **Bubalus bubalis** | [XP_006071188.1](https://www.ncbi.nlm.nih.gov/protein/XP_006071188.1) | [XP_025145302.1](https://www.ncbi.nlm.nih.gov/protein/XP_025145302.1) | [XP_006071186.1](https://www.ncbi.nlm.nih.gov/protein/XP_006071186.1) | [XP_006071184.1](https://www.ncbi.nlm.nih.gov/protein/XP_006071184.1) |
| **Camelus ferus** | [XP_014418049.1](https://www.ncbi.nlm.nih.gov/protein/XP_014418049.1) | [XP_006188732.1](https://www.ncbi.nlm.nih.gov/protein/XP_006188732.1) | [XP_032314538.1](https://www.ncbi.nlm.nih.gov/protein/XP_032314538.1) | [XP_006188734.1](https://www.ncbi.nlm.nih.gov/protein/XP_006188734.1) |
| **Capra hircus** | [XP_017904616.1](https://www.ncbi.nlm.nih.gov/protein/XP_017904616.1) | [XP_013820128.2](https://www.ncbi.nlm.nih.gov/protein/XP_013820128.2) | [XP_013820153.1](https://www.ncbi.nlm.nih.gov/protein/XP_013820153.1) | [NP_001272516.1](https://www.ncbi.nlm.nih.gov/protein/NP_001272516.1) |
| **Equus caballus** | [XP_023492613.1](https://www.ncbi.nlm.nih.gov/protein/XP_023492613.1) | [XP_014594001.1](https://www.ncbi.nlm.nih.gov/protein/XP_014594001.1) | [NP_001075321.1](https://www.ncbi.nlm.nih.gov/protein/NP_001075321.1) | [NP_001075353.1](https://www.ncbi.nlm.nih.gov/protein/NP_001075353.1) |
| **Ovis aries** | [XP_012034747.1](https://www.ncbi.nlm.nih.gov/protein/XP_012034747.1) | [NP_001009363.1](https://www.ncbi.nlm.nih.gov/protein/NP_001009363.1) | [NP_001009373.1](https://www.ncbi.nlm.nih.gov/protein/NP_001009373.1) | [NP_001009378.1](https://www.ncbi.nlm.nih.gov/protein/NP_001009378.1) |

**Table S2.** Percentage of sequence homology in representative species

|  | **Bos indicus** | **Bos indicus X Bos taurus** | **Bos mutus** | **Bos taurus** | **Bubalus bubalis** | **Camelus ferus** | **Capra hircus** | **Equus caballus** | **Ovis aries** |
| --- | --- | --- | --- | --- | --- | --- | --- | --- | --- |
| **Bos indicus** |  | 81% | 95% | 91% | 90% | 57% | 84% | 52% | 89% |
| **Bos indicus X Bos taurus** | 81% |  | 78% | 75% | 74% | 47% | 69% | 43% | 72% |
| **Bos mutus** | 95% | 78% |  | 95% | 93% | 55% | 87% | 50% | 85% |
| **Bos taurus** | 91% | 75% | 95% |  | 91% | 53% | 83% | 48% | 82% |
| **Bubalus bubalis** | 90% | 74% | 93% | 91% |  | 55% | 86% | 50% | 84% |
| **Camelus ferus** | 57% | 47% | 55% | 53% | 55% |  | 55% | 55% | 57% |
| **Capra hircus** | 84% | 69% | 87% | 83% | 86% | 55% |  | 50% | 93% |
| **Equus caballus** | 52 | 43% | 50% | 48% | 50% | 55% | 50% |  | 52% |
| **Ovis aries** | 89% | 72% | 85% | 82% | 84% | 57% | 93% | 52% |  |

**Table S3.** Putative transcription binding site and repressor site detected in Mediterranean buffalo

| **Gene** | **Transcription factor matrix ID (TRANSFAC R.3.4)** | **Transcription factor**  **(TRANSFAC R.3.4)** | **Similarity**  **(0.0-1.0)** | **Position on the input sequence** | **Forward (+) and reverse (-) strands with which transcription factor binds** | **Consensus**  **sequence** | **Sequence of the transcription factor binding sites** |
| --- | --- | --- | --- | --- | --- | --- | --- |
| **CSN1S1** | M00075 | V$GATA1_01 | 0.831688 | 5900 | (+) | SNNGA  TNNNN | ACAGA  TATCT |
|  | M00076 | V$GATA2_01 | 0.889039 | 5900 | (+) | NNNGA  TRNNN | ACAGA  TATCT |
|  | M00126 | V$GATA1_02 | 0.811250 | 5900 | (-) | NNNNNG  ATANKGNN | ACAGAT  ATCTCTAA |
|  | M00128 | V$GATA1_04 | 0.835784 | 5900 | (-) | NNCWGA  TARNNNN | ACAGAT  ATCTCTA |
|  | M00075 | V$GATA1_01 | 0.839585 | 5902 | (-) | SNNGAT  NNNN | AGATATCTCT |
|  | M00076 | V$GATA2_01 | 0.889039 | 5902 | (-) | NNNGAT  RNNN | AGATATCTCT |
|  | M00077 | V$GATA3_01 | 0.836066 | 5902 | (-) | NNGATARNG | AGATATCTC |
|  | M00162 | V$OCT1_06 | 0.821094 | 5903 | (-) | CWNAWTKWSATRYN | GATATCTCTAATTC |
|  | M00138 | V$OCT1_04 | 0.825178 | 5905 | (+) | NNNNNNNWATGCAAATNNNWNNW | TATCTCTAATTCAAAGAAAAAAA |
|  | M00210 | V$OCT_C | 0.768174 | 5911 | (-) | CTNATTTGCATAY | TAATTCAAAGAAA |
|  | M00223 | V$STAT_01 | 0.826821 | 5914 | (-) | TTCCCRKAA | TTCAAAGAA |
|  | M00216 | V$TATA_C | 0.755743 | 5919 | (+) | NCTATAAAAR | AGAAAAAAAG |
|  | M00075 | V$GATA1_01 | 0.774432 | 5929 | (-) | SNNGATNNNN | AAGCAACATG |
|  | M00059 | V$YY1_01 | 0.779161 | 5929 | (+) | NNNNNCCATNTWNNNWN | AAGCAACATGTATGTTG |
|  | M00162 | V$OCT1_06 | 0.804297 | 5948 | (+) | CWNAWTKWSATRYN | CAAAATTGGCTGGA |
|  | M00127 | V$GATA1_03 | 0.888045 | 5955 | (+) | RNSNNGATAANNGN | GGCTGGATAACTAA |
|  | M00075 | V$GATA1_01 | 0.821323 | 5957 | (+) | SNNGATNNNN | CTGGATAACT |
|  | M00076 | V$GATA2_01 | 0.840325 | 5957 | (+) | NNNGATRNNN | CTGGATAACT |
|  | M00203 | V$GATA_C | 0.882883 | 5959 | (+) | NGATAAGNMNN | GGATAACTAAA |
|  | M00076 | V$GATA2_01 | 0.802436 | 5971 | (+) | NNNGATRNNN | CAACATAGTA |
|  | M00216 | V$TATA_C | 0.754423 | 5986 | (+) | NCTATAAAAR | AGCTTAAATT |
|  | M00162 | V$OCT1_06 | 0.814062 | 5990 | (-) | CWNAWTKWSATRYN | TAAATTCTATGGAG |
|  | M00075 | V$GATA1_01 | 0.772458 | 6828 | (-) | SNNGATNNNN | AAGAATCTAT |
|  | M00162 | V$OCT1_06 | 0.834375 | 6829 | (-) | CWNAWTKWSATRYN | AGAATCTATTGCAG |
|  | M00137 | V$OCT1_03 | 0.847886 | 6839 | (+) | NNNRTAATNANNN | GCAGCAATTATGT |
|  | M00076 | V$GATA2_01 | 0.789355 | 6851 | (+) | NNNGATRNNN | TAGGATCAAT |
|  | M00162 | V$OCT1_06 | 0.813672 | 6855 | (+) | CWNAWTKWSATRYN | ATCAATGGGCTATG |
|  | M00076 | V$GATA2_01 | 0.861976 | 6862 | (-) | NNNGATRNNN | GGCTATGCTG |
|  | M00162 | V$OCT1_06 | 0.808594 | 6881 | (+) | CWNAWTKWSATRYN | CGCTCAGTCATGCC |
|  | M00076 | V$GATA2_01 | 0.799278 | 6886 | (-) | NNNGATRNNN | AGTCATGCCC |
|  | M00162 | V$OCT1_06 | 0.813672 | 9400 | (+) | CWNAWTKWSATRYN | CTCCATGGACTATA |
|  | M00162 | V$OCT1_06 | 0.890625 | 9431 | (+) | CWNAWTKWSATRYN | GTTCATGGGATTTT |
|  | M00075 | V$GATA1_01 | 0.805035 | 9436 | (+) | SNNGATNNNN | TGGGATTTTC |
|  | M00076 | V$GATA2_01 | 0.848895 | 9436 | (+) | NNNGATRNNN | TGGGATTTTC |
|  | M00225 | V$STAT3_01 | 0.718030 | 9437 | (+) | NGNNATTTCCSGGAARTGNNN | GGGATTTTCCAGGCAACATTT |
|  | M00223 | V$STAT_01 | 0.832871 | 9443 | (+) | TTCCCRKAA | TTCCAGGCA |
|  | M00223 | V$STAT_01 | 0.856819 | 9443 | (-) | TTCCCRKAA | TTCCAGGCA |
|  | M00162 | V$OCT1_06 | 0.855078 | 9450 | (+) | CWNAWTKWSATRYN | CAACATTTCCTTCT |
|  | M00162 | V$OCT1_06 | 0.876172 | 9451 | (-) | CWNAWTKWSATRYN | AACATTTCCTTCTG |
|  | M00075 | V$GATA1_01 | 0.825765 | 9467 | (+) | SNNGATNNNN | GCGGATCTTC |
|  | M00075 | V$GATA1_01 | 0.843040 | 9467 | (-) | SNNGATNNNN | GCGGATCTTC |
|  | M00076 | V$GATA2_01 | 0.863329 | 9467 | (+) | NNNGATRNNN | GCGGATCTTC |
|  | M00076 | V$GATA2_01 | 0.858367 | 9467 | (-) | NNNGATRNNN | GCGGATCTTC |
|  | M00137 | V$OCT1_03 | 0.862900 | 9482 | (+) | NNNRTAATNANNN | CCAGTAATCAAAC |
|  | M00128 | V$GATA1_04 | 0.810662 | 12509 | (-) | NNCWGATARNNNN | TTTCTTCTCAGGT |
|  | M00223 | V$STAT_01 | 0.826821 | 12510 | (+) | TTCCCRKAA | TTCTTCTCA |
|  | M00223 | V$STAT_01 | 0.809680 | 12555 | (-) | TTCCCRKAA | TTGTAGTAA |
|  | M00127 | V$GATA1_03 | 0.791524 | 12557 | (-) | RNSNNGATAANNGN | GTAGTAATCAATCT |
|  | M00137 | V$OCT1_03 | 0.873173 | 12557 | (+) | NNNRTAATNANNN | GTAGTAATCAATC |
|  | M00075 | V$GATA1_01 | 0.823791 | 12563 | (-) | SNNGATNNNN | ATCAATCTGA |
|  | M00076 | V$GATA2_01 | 0.834461 | 12563 | (-) | NNNGATRNNN | ATCAATCTGA |
|  | M00077 | V$GATA3_01 | 0.835623 | 12563 | (-) | NNGATARNG | ATCAATCTG |
|  | M00128 | V$GATA1_04 | 0.810662 | 12567 | (+) | NNCWGATARNNNN | ATCTGAGAAAGGA |
|  | M00223 | V$STAT_01 | 0.822536 | 12567 | (-) | TTCCCRKAA | ATCTGAGAA |
|  | M00138 | V$OCT1_04 | 0.794856 | 12579 | (-) | NNNNNNNWATGCAAA  TNNNWNNW | AGAACTATTTGTCATTCTTCTTT |
|  | M00162 | V$OCT1_06 | 0.929688 | 12583 | (+) | CWNAWTKWSATRYN | CTATTTGTCATTCT |
|  | M00162 | V$OCT1_06 | 0.862891 | 12584 | (-) | CWNAWTKWSATRYN | TATTTGTCATTCTT |
|  | M00127 | V$GATA1_03 | 0.808672 | 14803 | (+) | RNSNNGATAANNGN | ACTTTGATAGCTGG |
|  | M00075 | V$GATA1_01 | 0.850938 | 14805 | (+) | SNNGATNNNN | TTTGATAGCT |
|  | M00076 | V$GATA2_01 | 0.857014 | 14805 | (+) | NNNGATRNNN | TTTGATAGCT |
|  | M00059 | V$YY1_01 | 0.815203 | 14805 | (-) | NNNNNCCATNTWNNNWN | TTTGATAGCTGGACCTG |
|  | M00077 | V$GATA3_01 | 0.831192 | 14806 | (+) | NNGATARNG | TTGATAGCT |
|  | M00138 | V$OCT1_04 | 0.795065 | 14822 | (+) | NNNNNNNWATGCAA  ATNNNWNNW | TGTTAGTAATGCAGGTTCTTGGA |
|  | M00223 | V$STAT_01 | 0.812957 | 14837 | (+) | TTCCCRKAA | TTCTTGGAG |
|  | M00138 | V$OCT1_04 | 0.797783 | 14872 | (-) | NNNNNNNWATGCAAATNNNWNNW | AAGTGATTTATTCATTACCCCTC |
|  | M00075 | V$GATA1_01 | 0.797137 | 14873 | (+) | SNNGATNNNN | AGTGATTTAT |
|  | M00137 | V$OCT1_03 | 0.921375 | 14880 | (-) | NNNRTAATNANNN | TATTCATTACCCC |
|  | M00075 | V$GATA1_01 | 0.807996 | 14888 | (-) | SNNGATNNNN | ACCCCTCAGG |
|  | M00223 | V$STAT_01 | 0.798840 | 14897 | (+) | GTACCCTAA | TTCCCRKAA |
|  | M00216 | V$TATA_C | 0.744125 | 14901 | (+) | NCTATAAAAR | CCTAAGAAAA |
| **CSN1S2** | M00059 | V$YY1_01 | 0.792595 | 1000 | (-) | NNNNNCCATNTWNNNWN | AAGAATATCTGGGATTC |
|  | M00126 | V$GATA1_02 | 0.804688 | 1000 | (-) | NNNNNGATANKGNN | AAGAATATCTGGGA |
|  | M00075 | V$GATA1_01 | 0.885982 | 1002 | (-) | SNNGATNNNN | GAATATCTGG |
|  | M00076 | V$GATA2_01 | 0.907984 | 1002 | (-) | NNNGATRNNN | GAATATCTGG |
|  | M00077 | V$GATA3_01 | 0.828977 | 1002 | (-) | NNGATARNG | GAATATCTG |
|  | M00162 | V$OCT1_06 | 0.869922 | 1004 | (+) | CWNAWTKWSATRYN | ATATCTGGGATTCT |
|  | M00075 | V$GATA1_01 | 0.841066 | 1009 | (+) | SNNGATNNNN | TGGGATTCTT |
|  | M00076 | V$GATA2_01 | 0.863780 | 1009 | (+) | NNNGATRNNN | TGGGATTCTT |
|  | M00138 | V$OCT1_04 | 0.827269 | 1019 | (+) | NNNNNNNWATGCAAA  TNNNWNNW | TAGTAGGAATGATAAATTAATAA |
|  | M00126 | V$GATA1_02 | 0.799063 | 1024 | (+) | NNNNNGATANKGNN | GGAATGATAAATTA |
|  | M00127 | V$GATA1_03 | 0.831210 | 1024 | (+) | RNSNNGATAANNGN | GGAATGATAAATTA |
|  | M00128 | V$GATA1_04 | 0.849265 | 1025 | (+) | NNCWGATARNNNN | GAATGATAAATTA |
|  | M00075 | V$GATA1_01 | 0.773939 | 1026 | (+) | SNNGATNNNN | AATGATAAAT |
|  | M00076 | V$GATA2_01 | 0.795670 | 1026 | (+) | NNNGATRNNN | AATGATAAAT |
|  | M00077 | V$GATA3_01 | 0.823217 | 1027 | (+) | NNGATARNG | ATGATAAAT |
|  | M00203 | V$GATA_C | 0.893445 | 1028 | (+) | NGATAAGNMNN | TGATAAATTAA |
|  | M00162 | V$OCT1_06 | 0.831641 | 1037 | (-) | CWNAWTKWSATRYN | AATAACAAAAGCAG |
|  | M00135 | V$OCT1_01 | 0.764784 | 1048 | (+) | NNNNWTATGCAAA  TNTNNN | CAGGCAATGCTAATCTTTT |
|  | M00076 | V$GATA2_01 | 0.788453 | 1050 | (-) | NNNGATRNNN | GGCAATGCTA |
|  | M00195 | V$OCT1_Q6 | 0.817959 | 1050 | (+) | NNNNATGCAAATNAN | GGCAATGCTAATCTT |
|  | M00248 | V$OCT1_07 | 0.814925 | 1051 | (+) | TNTATGNTAATT | GCAATGCTAATC |
|  | M00210 | V$OCT_C | 0.814822 | 1052 | (-) | CTNATTTGCATAY | CAATGCTAATCTT |
|  | M00127 | V$GATA1_03 | 0.791524 | 1054 | (-) | RNSNNGATAANNGN | ATGCTAATCTTTTT |
|  | M00076 | V$GATA2_01 | 0.785295 | 1056 | (-) | NNNGATRNNN | GCTAATCTTT |
|  | M00077 | V$GATA3_01 | 0.853788 | 1056 | (-) | NNGATARNG | GCTAATCTT |
|  | M00138 | V$OCT1_04 | 0.804266 | 1058 | (-) | NNNNNNNWATGCAA  ATNNNWNNW | TAATCTTTTTTTAATTTAATTTT |
|  | M00162 | V$OCT1_06 | 0.908984 | 1062 | (+) | CWNAWTKWSATRYN | CTTTTTTTAATTTA |
|  | M00138 | V$OCT1_04 | 0.795483 | 1064 | (-) | NNNNNNNWATGCAA  ATNNNWNNW | TTTTTTAATTTAATTTTATTTTA |
|  | M00162 | V$OCT1_06 | 0.860547 | 1067 | (+) | CWNAWTKWSATRYN | TTTAATTTAATTTT |
|  | M00248 | V$OCT1_07 | 0.831673 | 1067 | (+) | TNTATGNTAATT | TTTAATTTAATT |
|  | M00162 | V$OCT1_06 | 0.804297 | 1072 | (+) | CWNAWTKWSATRYN | TTTAATTTTATTTT |
|  | M00248 | V$OCT1_07 | 0.831673 | 1078 | (+) | TNTATGNTAATT | TTTATTTTATTT |
|  | M00216 | V$TATA_C | 0.750726 | 1087 | (+) | NCTATAAAAR | TTTTTAAACT |
|  | M00138 | V$OCT1_04 | 0.804893 | 1088 | (-) | NNNNNNNWATGCAAATNNNWNNW | TTTTAAACTTTACAATATTGTAT |
|  | M00162 | V$OCT1_06 | 0.847266 | 1093 | (-) | CWNAWTKWSATRYN | AACTTTACAATATT |
|  | M00216 | V$TATA_C | 0.818326 | 1094 | (+) | NCTATAAAAR | ACTTTACAAT |
|  | M00136 | V$OCT1_02 | 0.840823 | 7401 | (+) | NNGAATATKCANNNN | CTGAATTTTCAATTT |
|  | M00162 | V$OCT1_06 | 0.821484 | 7403 | (+) | CWNAWTKWSATRYN | GAATTTTCAATTTA |
|  | M00162 | V$OCT1_06 | 0.826562 | 7404 | (-) | CWNAWTKWSATRYN | AATTTTCAATTTAT |
|  | M00162 | V$OCT1_06 | 0.808594 | 7412 | (+) | CWNAWTKWSATRYN | ATTTATTGAATCTC |
|  | M00162 | V$OCT1_06 | 0.833984 | 7420 | (-) | CWNAWTKWSATRYN | AATCTCTAATAAAC |
|  | M00216 | V$TATA_C | 0.752839 | 7422 | (+) | NCTATAAAAR | TCTCTAATAA |
|  | M00162 | V$OCT1_06 | 0.808203 | 7438 | (-) | CWNAWTKWSATRYN | TAACTCCAATATTT |
|  | M00076 | V$GATA2_01 | 0.785295 | 7458 | (-) | NNNGATRNNN | CCTCATGCGA |
|  | M00126 | V$GATA1_02 | 0.860000 | 7489 | (+) | NNNNNGATANKGNN | ACCCTGATACTGGG |
|  | M00127 | V$GATA1_03 | 0.885350 | 7489 | (+) | RNSNNGATAANNGN | ACCCTGATACTGGG |
|  | M00075 | V$GATA1_01 | 0.954097 | 7491 | (+) | SNNGATNNNN | CCTGATACTG |
|  | M00076 | V$GATA2_01 | 0.924673 | 7491 | (+) | NNNGATRNNN | CCTGATACTG |
|  | M00077 | V$GATA3_01 | 0.853345 | 7492 | (+) | NNGATARNG | CTGATACTG |
|  | M00162 | V$OCT1_06 | 0.847266 | 10307 | (-) | CWNAWTKWSATRYN | TATTTGTAAGTTAT |
|  | M00138 | V$OCT1_04 | 0.803848 | 10311 | (+) | NNNNNNNWATGCAAATNNNWNNW | TGTAAGTTATTTAAAAATATTAT |
|  | M00248 | V$OCT1_07 | 0.797329 | 10311 | (+) | TNTATGNTAATT | TGTAAGTTATTT |
|  | M00138 | V$OCT1_04 | 0.799875 | 10314 | (-) | NNNNNNNWATGCAA  ATNNNWNNW | AAGTTATTTAAAAATATTATTTT |
|  | M00162 | V$OCT1_06 | 0.839453 | 10316 | (+) | CWNAWTKWSATRYN | GTTATTTAAAAATA |
|  | M00195 | V$OCT1_Q6 | 0.794487 | 10316 | (-) | NNNNATGCAAATNAN | GTTATTTAAAAATAT |
|  | M00252 | V$TATA_01 | 0.797006 | 10317 | (+) | STATAAAWRNNNNNN | TTATTTAAAAATATT |
|  | M00216 | V$TATA_C | 0.848165 | 10318 | (+) | NCTATAAAAR | TATTTAAAAA |
|  | M00216 | V$TATA_C | 0.743597 | 10319 | (+) | NCTATAAAAR | ATTTAAAAAT |
|  | M00252 | V$TATA_01 | 0.793707 | 10319 | (+) | STATAAAWRNNNNNN | ATTTAAAAATATTAT |
|  | M00136 | V$OCT1_02 | 0.788576 | 10320 | (-) | NNGAATATKCANNNN | TTTAAAAATATTATT |
|  | M00127 | V$GATA1_03 | 0.811857 | 10343 | (-) | RNSNNGATAANNGN | GCACTTAACTCTGT |
|  | M00223 | V$STAT_01 | 0.870683 | 10356 | (+) | TTCCCRKAA | TTCCTCTCA |
|  | M00138 | V$OCT1_04 | 0.803220 | 10362 | (-) | NNNNNNNWATGCAAATNNNWNNW | TCAATTGTCTTGAATTCTTTCAA |
|  | M00162 | V$OCT1_06 | 0.808203 | 10374 | (+) | CWNAWTKWSATRYN | AATTCTTTCAATTA |
|  | M00162 | V$OCT1_06 | 0.803516 | 10388 | (+) | CWNAWTKWSATRYN | AGGCCTTCCATGTG |
|  | M00059 | V$YY1_01 0.778506 |  | 10390 | (+) | NNNNNCCATNTWNNNWN | GCCTTCCATGTGAACAA |
|  | M00075 | V$GATA1_01 | 0.820829 | 15100 | (-) | SNNGATNNNN | ATGAATCGCA |
|  | M00076 | V$GATA2_01 | 0.806044 | 15100 | (-) | NNNGATRNNN | ATGAATCGCA |
|  | M00075 | V$GATA1_01 | 0.866732 | 15105 | (-) | SNNGATNNNN | TCGCATCACA |
|  | M00076 | V$GATA2_01 | 0.850699 | 15105 | (-) | NNNGATRNNN | TCGCATCACA |
|  | M00075 | V$GATA1_01 | 0.917572 | 15127 | (-) | SNNGATNNNN | GTCCATCACC |
|  | M00076 | V$GATA2_01 | 0.870095 | 15127 | (-) | NNNGATRNNN | GTCCATCACC |
|  | M00075 | V$GATA1_01 | 0.814906 | 15133 | (-) | SNNGATNNNN | CACCAACTCC |
|  | M00224 | V$STAT1_01 | 0.748065 | 15133 | (+) | NNNSANTTCCGGGAANTGNSN | CACCAACTCCCGGAGTTCACT |
|  | M00224 | V$STAT1_01 | 0.766178 | 15133 | (-) | NNNSANTTCCGGGAANTGNSN | CACCAACTCCCGGAGTTCACT |
|  | M00225 | V$STAT3_01 | 0.717451 | 15133 | (+) | NGNNATTTCCSGGAARTGNNN | CACCAACTCCCGGAGTTCACT |
|  | M00225 | V$STAT3_01 | 0.768139 | 15133 | (-) | NGNNATTTCCSGGAARTGNNN | CACCAACTCCCGGAGTTCACT |
|  | M00075 | V$GATA1_01 | 0.791214 | 15154 | (-) | SNNGATNNNN | CACACTCACG |
|  | M00069 | V$YY1_02 | 0.778829 | 15159 | (+) | NNNCGGCCATCTT  GNCTSNW | TCACGTCCATCGAGTCAGTG |
|  | M00059 | V$YY1_01 | 0.818807 | 15160 | (+) | NNNNNCCATNTWNNNWN | CACGTCCATCGAGTCAG |
|  | M00075 | V$GATA1_01 | 0.851431 | 15163 | (-) | SNNGATNNNN | GTCCATCGAG |
|  | M00076 | V$GATA2_01 | 0.783040 | 15163 | (-) | NNNGATRNNN | GTCCATCGAG |
|  | M00075 | V$GATA1_01 | 0.846002 | 15175 | (+) | SNNGATNNNN | AGTGATGCCA |
|  | M00076 | V$GATA2_01 | 0.830853 | 15175 | (+) | NNNGATRNNN | AGTGATGCCA |
|  | M00069 | V$YY1_02 | 0.788647 | 15176 | (+) | NNNCGGCCATCTT  GNCTSNW | GTGATGCCATCCAGCCATCT |
|  | M00059 | V$YY1_01 | 0.801114 | 15177 | (+) | NNNNNCCATNT  WNNNWN | TGATGCCATCCAGCCAT |
|  | M00075 | V$GATA1_01 | 0.850938 | 15180 | (-) | SNNGATNNNN | TGCCATCCAG |
|  | M00076 | V$GATA2_01 | 0.833559 | 15180 | (-) | NNNGATRNNN | TGCCATCCAG |
|  | M00075 | V$GATA1_01 | 0.894867 | 15188 | (-) | SNNGATNNNN | AGCCATCTCA |
|  | M00076 | V$GATA2_01 | 0.929635 | 15188 | (-) | NNNGATRNNN | AGCCATCTCA |
|  | M00077 | V$GATA3_01 | 0.842712 | 15188 | (-) | NNGATARNG | AGCCATCTC |
|  | M00075 | V$GATA1_01 | 0.829714 | 15193 | (-) | SNNGATNNNN | TCTCATCCTC |
|  | M00076 | V$GATA2_01 | 0.856563 | 15193 | (-) | NNNGATRNNN | TCTCATCCTC |
|  | M00077 | V$GATA3_01 | 0.820558 | 15193 | (-) | NNGATARNG | TCTCATCCT |
| **CSN2** | M00059 | V$YY1_01 | 0.783093 | 3512 | (-) | NNNNNCCATNTWNNNWN | AGAAGGAAATGGCAACC |
|  | M00059 | V$YY1_01 | 0.839122 | 3528 | (+) | NNNNNCCATNTW  NNNWN | CCACTCCAGTTTTCTTG |
|  | M00127 | V$GATA1_03 | 0.837335 | 3533 | (-) | RNSNNGATAANNGN | CCAGTTTTCTTGCC |
|  | M00224 | V$STAT1_01 | 0.740655 | 3548 | (+) | NNNSANTTCCGGGAANTGNSN | GGAGAATCCCAGGGACTGGGG |
|  | M00075 | V$GATA1_01 | 0.826259 | 3549 | (-) | SNNGATNNNN | GAGAATCCCA |
|  | M00076 | V$GATA2_01 | 0.860171 | 3549 | (-) | NNNGATRNNN | GAGAATCCCA |
|  | M00162 | V$OCT1_06 | 0.857031 | 3550 | (-) | CWNAWTKWSATRYN | AGAATCCCAGGGAC |
|  | M00059 | V$YY1_01 0.784731 |  | 3581 | (-) | NNNNNCCATNT  WNNNWN | TGCCGTCTATGGGGTCA |
|  | M00076 | V$GATA2_01 | 0.793415 | 3585 | (-) | NNNGATRNNN | GTCTATGGGG |
|  | M00216 | V$TATA_C | 0.812252 | 6774 | (+) | NCTATAAAAR | CCCACAAAAC |
|  | M00162 | V$OCT1_06 | 0.834766 | 6829 | (+) | CWNAWTKWSATRYN | CAGCCTGAAATAAT |
|  | M00162 | V$OCT1_06 | 0.875781 | 6830 | (-) | CWNAWTKWSATRYN | AGCCTGAAATAATG |
|  | M00059 | V$YY1_01 0.807339 |  | 6833 | (-) | NNNNNCCATNTWNNNWN | CTGAAATAATGGGAGTC |
|  | M00137 | V$OCT1_03 | 0.853813 | 6835 | (+) | NNNRTAATNANNN | GAAATAATGGGAG |
|  | M00162 | V$OCT1_06 | 0.834375 | 6837 | (+) | CWNAWTKWSATRYN | AATAATGGGAGTCT |
|  | M00076 | V$GATA2_01 | 0.811006 | 6864 | (-) | NNNGATRNNN | GGCTATGGCT |
|  | M00162 | V$OCT1_06 | 0.863281 | 6880 | (+) | CWNAWTKWSATRYN | CACAAAGAAATGCC |
|  | M00224 | V$STAT1_01 | 0.732752 | 6889 | (-) | NNNSANTTCCGGGAANTGNSN | ATGCCCTTCCCTAAATATCCA |
|  | M00224 | V$STAT1_01 | 0.717457 | 6889 | (-) | NNNSANTTCCGGGAANTGNSN | ATGCCCTTCCCTAAATATCCA |
|  | M00162 | V$OCT1_06 | 0.813672 | 6894 | (+) | CWNAWTKWSATRYN | CTTCCCTAAATATC |
|  | M00223 | V$STAT_01 | 0.794807 | 6895 | (+) | TTCCCRKAA | TTCCCTAAA |
|  | M00136 | V$OCT1_02 | 0.786952 | 6896 | (-) | NNGAATATKCANNNN | TCCCTAAATATCCAG |
|  | M00127 | V$GATA1_03 | 0.784664 | 6899 | (-) | RNSNNGATAANNGN | CTAAATATCCAGTT |
|  | M00136 | V$OCT1_02 | 0.801841 | 6899 | (+) | NNGAATATKCANNNN | CTAAATATCCAGTTG |
| **CSN3** | M00076 | V$GATA2_01 | 0.797474 | 4105 | (-) | NNNGATRNNN | ACTTATGGGC |
|  | M00223 | V$STAT_01 | 0.813965 | 4107 | (+) | TTCCCRKAA | TTATGGGCA |
|  | M00223 | V$STAT_01 | 0.859340 | 4107 | (-) | TTCCCRKAA | TTATGGGCA |
|  | M00075 | V$GATA1_01 | 0.814906 | 5600 | (+) | SNNGATNNNN | GGAGTTGGTG |
|  | M00075 | V$GATA1_01 | 0.852419 | 5606 | (+) | SNNGATNNNN | GGTGATGAAC |
|  | M00076 | V$GATA2_01 | 0.818674 | 5606 | (+) | NNNGATRNNN | GGTGATGAAC |
|  | M00162 | V$OCT1_06 | 0.803125 | 5620 | (+) | CWNAWTKWSATRYN | AAGCCTGGCGTGCT |
|  | M00075 | V$GATA1_01 | 0.831194 | 5642 | (+) | SNNGATNNNN | TGGGATTGCA |
|  | M00076 | V$GATA2_01 | 0.878214 | 5642 | (+) | NNNGATRNNN | TGGGATTGCA |
|  | M00077 | V$GATA3_01 | 0.849358 | 5643 | (+) | NNGATARNG | GGGATTGCA |
|  | M00195 | V$OCT1_Q6 | 0.795852 | 5643 | (+) | NNNNATGCAAATNAN | GGGATTGCAAAGATC |
|  | M00075 | V$GATA1_01 | 0.788746 | 5651 | (-) | SNNGATNNNN | AAAGATCTGA |
|  | M00076 | V$GATA2_01 | 0.838069 | 5651 | (-) | NNNGATRNNN | AAAGATCTGA |
|  | M00138 | V$OCT1_04 | 0.782309 | 5653 | (-) | NNNNNNNWATGCAAATNNNWNNW | AGATCTGATATGACTAAGTAACT |
|  | M00126 | V$GATA1_02 | 0.798125 | 5654 | (+) | NNNNNGATANKGNN | GATCTGATATGACT |
|  | M00128 | V$GATA1_04 | 0.913297 | 5655 | (+) | NNCWGATARNNNN | ATCTGATATGACT |
|  | M00075 | V$GATA1_01 | 0.837611 | 5656 | (+) | SNNGATNNNN | TCTGATATGA |
|  | M00076 | V$GATA2_01 | 0.867839 | 5656 | (+) | NNNGATRNNN | TCTGATATGA |
|  | M00077 | V$GATA3_01 | 0.858219 | 5657 | (+) | NNGATARNG | CTGATATGA |
|  | M00203 | V$GATA_C | 0.886921 | 5658 | (+) | NGATAAGNMNN | TGATATGACTA |
|  | M00059 | V$YY1_01 0.781782 |  | 5666 | (-) | NNNNNCCATNTWNNNWN | CTAAGTAACTGAACTGA |
|  | M00223 | V$STAT_01 | 0.798840 | 5693 | (+) | TTCCCRKAA | TCCTCCTAA |
|  | M00162 | V$OCT1_06 | 0.828906 | 5700 | (-) | CWNAWTKWSATRYN | AAATTGTAAAATTT |
|  | 00216 | V$TATA_C | 0.777396 | 5702 | (+) | NCTATAAAAR | ATTGTAAAAT |

**Table S4.** Putative transcription binding site and repressor site detected in Swamp buffalo

| **Gene** | **Transcription factor matrix ID (TRANSFAC R.3.4)** | **Transcription factor**  **(TRANSFAC R.3.4)** | **Similarity**  **(0.0-1.0)** | **Position on the input sequence** | **Forward (+) and reverse (-) strands with which transcription factor binds** | **Consensus**  **sequence** | **Sequence of the transcription factor binding sites** |
| --- | --- | --- | --- | --- | --- | --- | --- |
| **CSN1S1** | M00136 | V$OCT1_02 | 0.841364 | 6501 | (-) | NNGAATATKCANNNN | ATAATGAATACTATG |
|  | M00076 | V$GATA2_01 | 0.804691 | 6509 | (-) | NNNGATRNNN | TACTATGAGC |
|  | M00059 | V$YY1_01 | 0.797837 | 6528 | (-) | NNNNNCCATNTWNNNWN | TATGAACAATGAACACT |
|  | M00069 | V$YY1_02 | 0.756159 | 6549 | (+) | NNNCGGCCATCTTGNCTSNW | GCAGGACCATATTGTTCTAA |
|  | M00059 | V$YY1_01 | 0.831258 | 6550 | (+) | NNNNNCCATNTWNNNWN | CAGGACCATATTGTTCT |
|  | M00076 | V$GATA2_01 | 0.811006 | 6553 | (+) | NNNGATRNNN | GACCATATTG |
|  | M00076 | V$GATA2_01 | 0.794317 | 6577 | (+) | NNNGATRNNN | GAGGTTACGG |
|  | M00128 | V$GATA1_04 | 0.817402 | 6585 | (-) | NNCWGATARNNNN | GGCCTAATCATAT |
|  | M00137 | V$OCT1_03 | 0.918214 | 6585 | (+) | NNNRTAATNANNN | GGCCTAATCATAT |
|  | M00059 | V$YY1_01 | 0.810944 | 6587 | (+) | NNNNNCCATNTWNNNWN | CCTAATCATATCATAAC |
|  | M00075 | V$GATA1_01 | 0.821816 | 6587 | (-) | SNNGATNNNN | CCTAATCATA |
|  | M00076 | V$GATA2_ 01 | 0.823184 | 6587 | (-) | NNNGATRNNN | CCTAATCATA |
|  | M00077 | V$GATA3_01 | 0.897209 | 6587 | (-) | NNGATARNG | CCTAATCAT |
|  | M00203 | V$GATA_C | 0.843119 | 6589 | (-) | NGATAAGNMNN | TAATCATATCA |
|  | M00126 | V$GATA1_02 | 0.819063 | 6590 | (-) | NNNNNGATANKGNN | AATCATATCATAAC |
|  | M00128 | V$GATA1_04 | 0.843137 | 6590 | (-) | NNCWGATARNNNN | AATCATATCATAA |
|  | M00075 | V$GATA1_01 | 0.816881 | 6592 | (-) | SNNGATNNNN | TCATATCATA |
|  | M00076 | V$GATA2_01 | 0.859720 | 6592 | (-) | NNNGATRNNN | TCATATCATA |
|  | M00077 | V$GATA3_01 | 0.863979 | 6592 | (-) | NNGATARNG | TCATATCAT |
|  | M00075 | V$GATA1_01 | 0.874630 | 8107 | (+) | SNNGATNNNN | CCTGATTTGA |
|  | M00076 | V$GATA2_01 | 0.812359 | 8107 | (+) | NNNGATRNNN | CCTGATTTGA |
|  | M00077 | V$GATA3_01 | 0.824546 | 8108 | (+) | NNGATARNG | CTGATTTGA |
|  | M00195 | V$OCT1_Q6 | 0.797216 | 8108 | (-) | NNNNATGCAAATNAN | CTGATTTGAAGAACT |
|  | M00210 | V$OCT_C | 0.774031 | 8108 | (+) | CTNATTTGCATAY | CTGATTTGAAGAA |
|  | M00223 | V$STAT_01 | 0.793043 | 8112 | (-) | TTCCCRKAA | TTTGAAGAA |
|  | M00075 | V$GATA1_01 | 0.834156 | 8120 | (+) | SNNGATNNNN | ACTGATGCAT |
|  | M00076 | V$GATA2_01 | 0.801534 | 8120 | (+) | NNNGATRNNN | ACTGATGCAT |
|  | M00075 | V$GATA1_01 | 0.954097 | 8140 | (+) | SNNGATNNNN | CCTGATGCTG |
|  | M00076 | V$GATA2_01 | 0.896256 | 8140 | (+) | NNNGATRNNN | CCTGATGCTG |
|  | M00223 | V$STAT_01 | 0.803126 | 8145 | (+) | TTCCCRKAA | TGCTGGGAA |
|  | M00223 | V$STAT_01 | 0.921099 | 8145 | (-) | TTCCCRKAA | TGCTGGGAA |
|  | M00077 | V$GATA3_01 | 0.873283 | 8153 | (+) | NNGATARNG | AAGATTGAA |
|  | M00127 | V$GATA1_03 | 0.797158 | 8162 | (+) | RNSNNGATAANNGN | GGCAGGAGAGGAGA |
|  | M00127 | V$GATA1_03 | 0.827536 | 8167 | (+) | RNSNNGATAANNGN | GAGAGGAGAAGAGG |
|  | M00127 | V$GATA1_03 | 0.796178 | 8175 | (+) | RNSNNGATAANNGN | AAGAGGACAAAGGA |
|  | M00076 | V$GATA2_01 | 0.809653 | 8184 | (+) | NNNGATRNNN | AAGGATGAGA |
|  | M00077 | V$GATA3_01 | 0.820558 | 8185 | (+) | NNGATARNG | AGGATGAGA |
|  | M00075 | V$GATA1_01 | 0.894867 | 8189 | (+) | SNNGATNNNN | TGAGATGGTT |
|  | M00076 | V$GATA2_01 | 0.910690 | 8189 | (+) | NNNGATRNNN | TGAGATGGTT |
|  | M00077 | V$GATA3_01 | 0.858662 | 8190 | (+) | NNGATARNG | GAGATGGTT |
|  | M00059 | V$YY1_01 | 0.785387 | 8193 | (-) | NNNNNCCATNTWNNNWN | ATGGTTGGATGGCATCA |
|  | M00075 | V$GATA1_01 | 0.790227 | 8197 | (+) | SNNGATNNNN | TTGGATGGCA |
|  | M00076 | V$GATA2_01 | 0.824538 | 8197 | (+) | NNNGATRNNN | TTGGATGGCA |
|  | M00059 | V$YY1_01 | 0.872870 | 8206 | (-) | NNNNNCCATNTWNNNWN | ATCACTGAATGGACATG |
|  | M00077 | V$GATA3_01 | 0.828977 | 15500 | (+) | NNGATARNG | CAGATATTT |
|  | M00162 | V$OCT1_06 | 0.808203 | 15501 | (+) | CWNAWTKWSATRYN | AGATATTTCCTTCT |
|  | M00203 | V$GATA_C | 0.849332 | 15501 | (+) | NGATAAGNMNN | AGATATTTCCT |
|  | M00162 | V$OCT1_06 | 0.829297 | 15502 | (-) | CWNAWTKWSATRYN | GATATTTCCTTCTT |
|  | M00162 | V$OCT1_06 | 0.942578 | 15510 | (+) | CWNAWTKWSATRYN | CTTCTTTTGATGCT |
|  | M00195 | V$OCT1_Q6 | 0.790120 | 15510 | (-) | NNNNATGCAAATNAN | CTTCTTTTGATGCTT |
|  | M00210 | V$OCT_C | 0.797052 | 15510 | (+) | CTNATTTGCATAY | CTTCTTTTGATGC |
|  | M00075 | V$GATA1_01 | 0.825271 | 15515 | (+) | SNNGATNNNN | TTTGATGCTT |
|  | M00076 | V$GATA2_01 | 0.784393 | 15515 | (+) | NNNGATRNNN | TTTGATGCTT |
|  | M00127 | V$GATA1_03 | 0.800833 | 15560 | (+) | RNSNNGATAANNGN | AGCTGGATTATTAG |
|  | M00075 | V$GATA1_01 | 0.797631 | 15562 | (+) | SNNGATNNNN | CTGGATTATT |
|  | M00075 | V$GATA1_01 | 0.830207 | 15572 | (+) | SNNGATNNNN | AGTGATCCTT |
|  | M00076 | V$GATA2_01 | 0.805593 | 15572 | (+) | NNNGATRNNN | AGTGATCCTT |
|  | M00076 | V$GATA2_01 | 0.815968 | 15572 | (-) | NNNGATRNNN | AGTGATCCTT |
|  | M00137 | V$OCT1_03 | 0.882655 | 15578 | (-) | NNNRTAATNANNN | CCTTCATTATTTA |
|  | M00162 | V$OCT1_06 | 0.847266 | 15583 | (+) | CWNAWTKWSATRYN | ATTATTTACAATTT |
|  | M00195 | V$OCT1_Q6 | 0.813865 | 15583 | (-) | NNNNATGCAAATNAN | ATTATTTACAATTTA |
|  | M00138 | V$OCT1_04 | 0.794019 | 15585 | (+) | NNNNNNNWATGCAAATNNNWNNW | TATTTACAATTTAGAAAATAGGA |
|  | M00216 | V$TATA_C | 0.776340 | 15594 | (+) | NCTATAAAAR | TTTAGAAAAT |
|  | M00126 | V$GATA1_02 | 0.785000 | 15596 | (+) | NNNNNGATANKGNN | TAGAAAATAGGAGT |
| **CSN1S2** | M00138 | V$OCT1_04 | 0.795483 | 1800 | (-) | NNNNNNNWATGCAAATNNNWNNW | TTTTTTAATTTAATTTTATTTTA |
|  | M00162 | V$OCT1_06 | 0.860547 | 1803 | (+) | CWNAWTKWSATRYN | TTTAATTTAATTTT |
|  | M00248 | V$OCT1_07 | 0.831673 | 1803 | (+) | TNTATGNTAATT | TTTAATTTAATT |
|  | M00162 | V$OCT1_06 | 0.804297 | 1808 | (+) | CWNAWTKWSATRYN | TTTAATTTTATTTT |
|  | M00248 | V$OCT1_07 | 0.831673 | 1814 | (+) | TNTATGNTAATT | TTTATTTTATTT |
|  | M00216 | V$TATA_C | 0.750726 | 1823 | (+) | NCTATAAAAR | TTTTTAAACT |
|  | M00138 | V$OCT1_04 | 0.804893 | 1824 | (-) | NNNNNNNWATGCAAATNNNWNNW | TTTTAAACTTTACAATATTGTAT |
|  | M00162 | V$OCT1_06 | 0.847266 | 1829 | (-) | CWNAWTKWSATRYN | AACTTTACAATATT |
|  | M00216 | V$TATA_C | 0.818326 | 1830 | (+) | NCTATAAAAR | ACTTTACAAT |
|  | M00059 | V$YY1_01 | 0.865334 | 1853 | (-) | NNNNNCCATNTWNNNWN | TGCCAAACATGGAAATG |
|  | M00075 | V$GATA1_01 | 0.773939 | 1854 | (-) | SNNGATNNNN | GCCAAACATG |
|  | M00135 | V$OCT1_01 | 0.765357 | 1855 | (+) | NNNNWTATGCAAATNTNNN | CCAAACATGGAAATGAATC |
|  | M00195 | V$OCT1_Q6 | 0.838701 | 1857 | (+) | NNNNATGCAAATNAN | AAACATGGAAATGAA |
|  | M00161 | V$OCT1_05 | 0.878632 | 1858 | (-) | MKNATTTGCATAYY | AACATGGAAATGAA |
|  | M00162 | V$OCT1_06 | 0.812109 | 1858 | (-) | CWNAWTKWSATRYN | AACATGGAAATGAA |
|  | M00248 | V$OCT1_07 | 0.795845 | 1858 | (+) | TNTATGNTAATT | AACATGGAAATG |
|  | M00210 | V$OCT_C | 0.863489 | 1859 | (-) | CTNATTTGCATAY | ACATGGAAATGAA |
|  | M00075 | V$GATA1_01 | 0.849951 | 1867 | (-) | SNNGATNNNN | ATGAATCCGC |
|  | M00076 | V$GATA2_01 | 0.838069 | 1867 | (-) | NNNGATRNNN | ATGAATCCGC |
|  | M00059 | V$YY1_01 | 0.803735 | 1892 | (+) | NNNNNCCATNTWNNNWN | GCTCCCCATCCTGAACC |
|  | M00075 | V$GATA1_01 | 0.946199 | 1895 | (-) | SNNGATNNNN | CCCCATCCTG |
|  | M00076 | V$GATA2_01 | 0.945422 | 1895 | (-) | NNNGATRNNN | CCCCATCCTG |
|  | M00077 | V$GATA3_01 | 0.883917 | 1895 | (-) | NNGATARNG | CCCCATCCT |
|  | M00069 | V$YY1_02 | 0.775616 | 1901 | (+) | NNNCGGCCATCTTGNCTSNW | CCTGAACCCTCCTCCCTCCT |
|  | M00069 | V$YY1_02 | 0.791860 | 5995 | (+) | NNNCGGCCATCTTGNCTSNW | TGAGAGCCATTTTTGAGCCA |
|  | M00059 | V$YY1_01 | 0.891874 | 5996 | (+) | NNNNNCCATNTWNNNWN | GAGAGCCATTTTTGAGC |
|  | M00136 | V$OCT1_02 | 0.778560 | 6023 | (-) | NNGAATATKCANNNN | AAGCAGAATATCTTT |
|  | M00126 | V$GATA1_02 | 0.813438 | 6026 | (-) | NNNNNGATANKGNN | CAGAATATCTTTGA |
|  | M00075 | V$GATA1_01 | 0.796150 | 6028 | (-) | SNNGATNNNN | GAATATCTTT |
|  | M00076 | V$GATA2_01 | 0.858367 | 6028 | (-) | NNNGATRNNN | GAATATCTTT |
|  | M00077 | V$GATA3_01 | 0.834736 | 6028 | (-) | NNGATARNG | GAATATCTT |
|  | M00216 | V$TATA_C | 0.852390 | 6033 | (+) | NCTATAAAAR | TCTTTGAAAG |
|  | M00162 | V$OCT1_06 | 0.862891 | 6040 | (+) | CWNAWTKWSATRYN | AAGAATTTCTTGTT |
|  | M00223 | V$STAT_01 | 0.807663 | 6046 | (+) | TTCCCRKAA | TTCTTGTTA |
|  | M00138 | V$OCT1_04 | 0.804057 | 6049 | (-) | NNNNNNNWATGCAAATNNNWNNW | TTGTTAAAGATTCATCATCTTTA |
|  | M00059 | V$YY1_01 | 0.788991 | 6058 | (+) | NNNNNCCATNTWNNNWN | ATTCATCATCTTTACTT |
|  | M00075 | V$GATA1_01 | 0.838598 | 6058 | (-) | SNNGATNNNN | ATTCATCATC |
|  | M00076 | V$GATA2_01 | 0.814163 | 6058 | (-) | NNNGATRNNN | ATTCATCATC |
|  | M00126 | V$GATA1_02 | 0.776875 | 6059 | (-) | NNNNNGATANKGNN | TTCATCATCTTTAC |
|  | M00075 | V$GATA1_01 | 0.804047 | 6061 | (-) | SNNGATNNNN | CATCATCTTT |
|  | M00076 | V$GATA2_01 | 0.831755 | 6061 | (-) | NNNGATRNNN | CATCATCTTT |
|  | M00077 | V$GATA3_01 | 0.879929 | 6061 | (-) | NNGATARNG | CATCATCTT |
|  | M00137 | V$OCT1_03 | 0.862505 | 6063 | (-) | NNNRTAATNANNN | TCATCTTTACTTT |
|  | M00162 | V$OCT1_06 | 0.842187 | 6064 | (+) | CWNAWTKWSATRYN | CATCTTTACTTTTC |
|  | M00127 | V$GATA1_03 | 0.834150 | 6069 | (-) | RNSNNGATAANNGN | TTACTTTTCTTCCT |
|  | M00216 | V$TATA_C | 0.748878 | 6101 | (+) | NCTATAAAAR | TTCACAAAAT |
|  | M00059 | V$YY1_01 | 0.846003 | 6105 | (+) | NNNNNCCATNTWNNNWN | CAAAATCATATTTTCTT |
|  | M00059 | V$YY1_01 | 0.870577 | 11090 | (-) | NNNNNCCATNTWNNNWN | ATTTCTATATGAAGCTA |
|  | M00248 | V$OCT1_07 | 0.901420 | 11103 | (+) | TNTATGNTAATT | GCTATTTTAATT |
|  | M00138 | V$OCT1_04 | 0.812631 | 11104 | (+) | NNNNNNNWATGCAAATNNNWNNW | CTATTTTAATTAATATTTTCAAA |
|  | M00138 | V$OCT1_04 | 0.847553 | 11105 | (-) | NNNNNNNWATGCAAATNNNWNNW | TATTTTAATTAATATTTTCAAAA |
|  | M00136 | V$OCT1_02 | 0.786140 | 11109 | (-) | NNGAATATKCANNNN | TTAATTAATATTTTC |
|  | M00138 | V$OCT1_04 | 0.814931 | 11111 | (-) | NNNNNNNWATGCAAATNNNWNNW | AATTAATATTTTCAAAATCTAAT |
|  | M00248 | V$OCT1_07 | 0.818105 | 11111 | (-) | TNTATGNTAATT | AATTAATATTTT |
|  | M00136 | V$OCT1_02 | 0.788847 | 11112 | (+) | NNGAATATKCANNNN | ATTAATATTTTCAAA |
|  | M00138 | V$OCT1_04 | 0.802175 | 11112 | (+) | NNNNNNNWATGCAAATNNNWNNW | ATTAATATTTTCAAAATCTAATA |
|  | M00135 | V$OCT1_01 | 0.770889 | 11113 | (-) | NNNNWTATGCAAATNTNNN | TTAATATTTTCAAAATCTA |
|  | M00210 | V$OCT_C | 0.765953 | 11115 | (+) | CTNATTTGCATAY | AATATTTTCAAAA |
|  | M00216 | V$TATA_C | 0.761025 | 11118 | (+) | NCTATAAAAR | ATTTTCAAAA |
|  | M00216 | V$TATA_C | 0.750726 | 11119 | (+) | NCTATAAAAR | TTTTCAAAAT |
|  | M00075 | V$GATA1_01 | 0.770977 | 11123 | (-) | SNNGATNNNN | CAAAATCTAA |
|  | M00077 | V$GATA3_01 | 0.843598 | 11123 | (-) | NNGATARNG | CAAAATCTA |
|  | M00162 | V$OCT1_06 | 0.873828 | 11123 | (+) | CWNAWTKWSATRYN | CAAAATCTAATATC |
|  | M00162 | V$OCT1_06 | 0.842187 | 11124 | (-) | CWNAWTKWSATRYN | AAAATCTAATATCA |
|  | M00138 | V$OCT1_04 | 0.801338 | 11125 | (-) | NNNNNNNWATGCAAATNNNWNNW | AAATCTAATATCAATAATTAGGC |
|  | M00126 | V$GATA1_02 | 0.865000 | 11128 | (-) | NNNNNGATANKGNN | TCTAATATCAATAA |
|  | M00248 | V$OCT1_07 | 0.791181 | 11131 | (-) | TNTATGNTAATT | AATATCAATAAT |
|  | M00137 | V$OCT1_03 | 0.877519 | 11135 | (+) | NNNRTAATNANNN | TCAATAATTAGGC |
|  | M00137 | V$OCT1_03 | 0.853023 | 11136 | (-) | NNNRTAATNANNN | CAATAATTAGGCT |
|  | M00135 | V$OCT1_01 | 0.759824 | 11138 | (+) | NNNNWTATGCAAATNTNNN | ATAATTAGGCTTATCTGAA |
|  | M00203 | V$GATA_C 0 | .961168 | 11143 | (-) | NGATAAGNMNN | TAGGCTTATCT |
|  | M00126 | V$GATA1_02 | 0.868437 | 11144 | (-) | NNNNNGATANKGNN | AGGCTTATCTGAAA |
|  | M00128 | V$GATA1_04 | 0.953431 | 11144 | (-) | NNCWGATARNNNN | AGGCTTATCTGAA |
|  | M00075 | V$GATA1_01 | 0.819348 | 11146 | (-) | SNNGATNNNN | GCTTATCTGA |
|  | M00076 | V$GATA2_01 | 0.890392 | 11146 | (-) | NNNGATRNNN | GCTTATCTGA |
|  | M00077 | V$GATA3_01 | 0.881701 | 11146 | (-) | NNGATARNG | GCTTATCTG |
|  | M00223 | V$STAT_01 | 0.809680 | 11148 | (+) | TTCCCRKAA | TTATCTGAA |
|  | M00223 | V$STAT_01 | 0.826569 | 11148 | (-) | TTCCCRKAA | TTATCTGAA |
|  | M00162 | V$OCT1_06 | 0.832031 | 11162 | (+) | CWNAWTKWSATRYN | CTGCTTTTAATAAA |
|  | M00210 | V$OCT_C | 0.797456 | 11162 | (+) | CTGCTTTTAATAA | CTNATTTGCATAY |
|  | M00137 | V$OCT1_03 | 0.852232 | 11165 | (+) | NNNRTAATNANNN | CTTTTAATAAAAT |
|  | M00216 | V$TATA_C | 0.767626 | 11165 | (+) | NCTATAAAAR | CTTTTAATAA |
|  | M00216 | V$TATA_C | 0.817798 | 11168 | (+) | NCTATAAAAR | TTAATAAAAT |
|  | M00162 | V$OCT1_06 | 0.847266 | 11185 | (-) | CWNAWTKWSATRYN | TATTTGTAAGTTAT |
|  | M00138 | V$OCT1_04 | 0.803848 | 11189 | (+) | NNNNNNNWATGCAAATNNNWNNW | TGTAAGTTATTTAAAAATATTAT |
|  | M00248 | V$OCT1_07 | 0.797329 | 11189 | (+) | TNTATGNTAATT | TGTAAGTTATTT |
|  | M00138 | V$OCT1_04 | 0.799875 | 11192 | (-) | NNNNNNNWATGCAAATNNNWNNW | AAGTTATTTAAAAATATTATTTT |
|  | M00162 | V$OCT1_06 | 0.839453 | 11194 | (+) | CWNAWTKWSATRYN | GTTATTTAAAAATA |
|  | M00195 | V$OCT1_Q6 | 0.794487 | 11194 | (-) | NNNNATGCAAATNAN | GTTATTTAAAAATAT |
|  | M00252 | V$TATA_01 | 0.797006 | 11195 | (+) | STATAAAWRNNNNNN | TTATTTAAAAATATT |
|  | M00216 | V$TATA_C | 0.848165 | 11196 | (+) | NCTATAAAAR | TATTTAAAAA |
|  | M00216 | V$TATA_C | 0.743597 | 11197 | (+) | NCTATAAAAR | ATTTAAAAAT |
|  | M00252 | V$TATA_01 | 0.793707 | 11198 | (+) | STATAAAWRNNNNNN | ATTTAAAAATATTAT |
|  | M00136 | $OCT1_02 | 0.788576 | 11198 | (-) | NNGAATATKCANNNN | TTTAAAAATATTATT |
|  | M00075 | V$GATA1_01 | 0.917572 | 16006 | (-) | SNNGATNNNN | GTCCATCACC |
|  | M00076 | V$GATA2_01 | 0.870095 | 16006 | (-) | NNNGATRNNN | GTCCATCACC |
|  | M00075 | V$GATA1_01 | 0.814906 | 16012 | (-) | SNNGATNNNN | CACCAACTCC |
|  | M00224 | V$STAT1_01 | 0.744278 | 16012 | (+) | NNNSANTTCCGGGAANTGNSN | CACCAACTCCCGGAGTTCACC |
|  | M00224 | V$STAT1_01 | 0.791866 | 16012 | (-) | NNNSANTTCCGGGAANTGNSN | CACCAACTCCCGGAGTTCACC |
|  | M00225 | V$STAT3_01 | 0.710500 | 16012 | (+) | NGNNATTTCCSGGAARTGNNN | CACCAACTCCCGGAGTTCACC |
|  | M00225 | V$STAT3_01 | 0.777118 | 16012 | (-) | NGNNATTTCCSGGAARTGNNN | CACCAACTCCCGGAGTTCACC |
|  | M00059 | V$YY1_01 | 0.840433 | 16039 | (+) | NNNNNCCATNTWNNNWN | CAAGTCCATCGAGTCAG |
|  | M00075 | V$GATA1_01 | 0.851431 | 16042 | (-) | SNNGATNNNN | GTCCATCGAG |
|  | M00076 | V$GATA2_01 | 0.783040 | 16042 | (-) | NNNGATRNNN | GTCCATCGAG |
|  | M00075 | V$GATA1_01 | 0.846002 | 16054 | (+) | SNNGATNNNN | AGTGATGCCA |
|  | M00076 | V$GATA2_01 | 0.830853 | 16054 | (+) | NNNGATRNNN | AGTGATGCCA |
|  | M00069 | V$YY1_02 | 0.788647 | 16055 | (+) | NNNCGGCCATCTTGNCTSNW | GTGATGCCATCCAGCCATCT |
|  | M00059 | V$YY1_01 | 0.801114 | 16056 | (+) | NNNNNCCATNTWNNNWN | TGATGCCATCCAGCCAT |
|  | M00075 | V$GATA1_01 | 0.850938 | 16059 | (-) | SNNGATNNNN | TGCCATCCAG |
|  | M00076 | V$GATA2_01 | 0.833559 | 16059 | (-) | NNNGATRNNN | TGCCATCCAG |
|  | M00075 | V$GATA1_01 | 0.894867 | 16067 | (-) | SNNGATNNNN | AGCCATCTCA |
|  | M00076 | V$GATA2_01 | 0.929635 | 16067 | (-) | NNNGATRNNN | AGCCATCTCA |
|  | M00077 | V$GATA3_01 | 0.842712 | 16067 | (-) | NNGATARNG | AGCCATCTC |
|  | M00075 | V$GATA1_01 | 0.829714 | 16072 | (-) | SNNGATNNNN | TCTCATCCTC |
|  | M00076 | V$GATA2_01 | 0.856563 | 16072 | (-) | NNNGATRNNN | TCTCATCCTC |
|  | M00077 | V$GATA3_01 | 0.820558 | 16072 | (-) | NNGATARNG | TCTCATCCT |
|  | M00075 | V$GATA1_01 | 0.802073 | 16088 | (-) | SNNGATNNNN | CCCCTTCTCC |
|  | M00127 | V$GATA1_03 | 0.870162 | 16088 | (-) | RNSNNGATAANNGN | CCCCTTCTCCTCCT |
| **CSN2** | M00059 | V$YY1_01 | 0.785387 | 2192 | (-) | NNNNNCCATNTWNNNWN | CTAAATAAATGTTACTT |
|  | M00127 | V$GATA1_03 | 0.796423 | 2202 | (-) | RNSNNGATAANNGN | GTTACTTTCTCTCT |
|  | M00216 | V$TATA_C | 0.752839 | 2211 | (+) | NCTATAAAAR | TCTCTTAAAA |
|  | M00216 | V$TATA_C | 0.844732 | 2212 | (+) | NCTATAAAAR | CTCTTAAAAT |
|  | M00075 | V$GATA1_01 | 0.775913 | 2216 | (-) | SNNGATNNNN | TAAAATCCCT |
|  | M00076 | V$GATA2_01 | 0.790257 | 2228 | (-) | NNNGATRNNN | TAAAATCCCT |
|  | M00075 | V$GATA1_01 | 0.888450 | 2228 | (-) | SNNGATNNNN | ACAAATCCCC |
|  | M00076 | V$GATA2_01 | 0.873252 | 2202 | (-) | NNNGATRNNN | ACAAATCCCC |
|  | M00126 | V$GATA1_02 | 0.929688 | 2235 | (-) | NNNNNGATANKGNN | CCCACTATCTAGAG |
|  | M00127 | V$GATA1_03 | 0.887310 | 2235 | (-) | RNSNNGATAANNGN | CCCACTATCTAGAG |
|  | M00128 | V$GATA1_04 | 0.856311 | 2235 | (-) | NNCWGATARNNNN | CCCACTATCTAGA |
|  | M00075 | V$GATA1_01 | 0.902270 | 2237 | (-) | SNNGATNNNN | CACTATCTAG |
|  | M00076 | V$GATA2_01 | 0.871448 | 2237 | (-) | NNNGATRNNN | CACTATCTAG |
|  | M00077 | V$GATA3_01 | 0.937528 | 2237 | (-) | NNGATARNG | CACTATCTA |
|  | M00127 | V$GATA1_03 | 0.810142 | 2241 | (+) | RNSNNGATAANNGN | ATCTAGAGAATAAG |
|  | M00075 | V$GATA1_01 | 0.788253 | 2251 | (+) | SNNGATNNNN | TAAGATTGAC |
|  | M00076 | V$GATA2_01 | 0.822733 | 2251 | (+) | NNNGATRNNN | TAAGATTGAC |
|  | M00077 | V$GATA3_01 | 0.841382 | 2252 | (+) | NNGATARNG | AAGATTGAC |
|  | M00162 | V$OCT1_06 | 0.919531 | 2252 | (+) | CWNAWTKWSATRYN | AAGATTGACATTCC |
|  | M00162 | V$OCT1_06 | 0.821484 | 2253 | (-) | CWNAWTKWSATRYN | AGATTGACATTCCC |
|  | M00224 | V$STAT1_01 | 0.730940 | 2256 | (+) | NNNSANTTCCGGGAANTGNSN | TTGACATTCCCTGGAGTCACA |
|  | M00162 | V$OCT1_06 | 0.808594 | 2258 | (-) | CWNAWTKWSATRYN | GACATTCCCTGGAG |
|  | M00059 | V$YY1_01 | 0.787025 | 2272 | (+) | NNNNNCCATNTWNNNWN | TCACAGCATGCTTTGTC |
|  | M00076 | V$GATA2_01 | 0.792963 | 2275 | (+) | NNNGATRNNN | CAGCATGCTT |
|  | M00203 | V$GATA_C | 0.873563 | 2279 | (-) | NGATAAGNMNN | ATGCTTTGTCT |
|  | M00162 | V$OCT1_06 | 0.855078 | 2285 | (-) | CWNAWTKWSATRYN | TGTCTGCCATTATC |
|  | M00059 | V$YY1_01 | 0.775557 | 2286 | (+) | NNNNNCCATNTWNNNWN | GTCTGCCATTATCTGAC |
|  | M00137 | V$OCT1_03 | 0.850652 | 2288 | (-) | NNNRTAATNANNN | CTGCCATTATCTG |
|  | M00203 | V$GATA_C | 0.889096 | 2289 | (-) | NGATAAGNMNN | TGCCATTATCT |
|  | M00126 | V$GATA1_02 | 0.868125 | 2290 | (-) | NNNNNGATANKGNN | GCCATTATCTGACC |
|  | M00127 | V$GATA1_03 | 0.869917 | 2290 | (-) | RNSNNGATAANNGN | GCCATTATCTGACC |
|  | M00128 | V$GATA1_04 | 0.942708 | 2290 | (-) | NNCWGATARNNNN | GCCATTATCTGAC |
|  | M00075 | V$GATA1_01 | 0.833169 | 2292 | (-) | SNNGATNNNN | CATTATCTGA |
|  | M00076 | V$GATA2_01 | 0.900767 | 2292 | (-) | NNNGATRNNN | CATTATCTGA |
|  | M00077 | V$GATA3_01 | 0.927780 | 2292 | (-) | NNGATARNG | CATTATCTG |
|  | M00059 | V$YY1_01 | 0.783093 | 6507 | (-) | NNNNNCCATNTWNNNWN | AGAAGGAAATGGCAACC |
|  | M00075 | V$GATA1_01 | 0.803554 | 6520 | (-) | SNNGATNNNN | AACCAACTCC |
|  | M00059 | V$YY1_01 | 0.849934 | 6523 | (+) | NNNNNCCATNTWNNNWN | CAACTCCAGTTTTCTTG |
|  | M00127 | V$GATA1_03 | 0.837335 | 6528 | (-) | RNSNNGATAANNGN | CCAGTTTTCTTGCC |
|  | M00224 | V$STAT1_01 | 0.740655 | 6543 | (+) | NNNSANTTCCGGGAANTGNSN | GGAGAATCCCAGGGACTGGGG |
|  | M00075 | V$GATA1_01 | 0.826259 | 6544 | (-) | SNNGATNNNN | GAGAATCCCA |
|  | M00076 | V$GATA2_01 | 0.860171 | 6544 | (-) | NNNGATRNNN | GAGAATCCCA |
|  | M00162 | V$OCT1_06 | 0.857031 | 6545 | (-) | CWNAWTKWSATRYN | AGAATCCCAGGGAC |
|  | M00059 | V$YY1_01 | 0.777851 | 6576 | (-) | NNNNNCCATNTWNNNWN | TGCCGTCTATGGGGTCG |
|  | M00076 | V$GATA2_01 | 0.793415 | 6580 | (-) | NNNGATRNNN | GTCTATGGGG |
| **CSN3** | M00075 | V$GATA1_01 | 0.776407 | 534 | (+) | SNNGATNNNN | GCTGACGGTC |
|  | M00127 | V$GATA1_03 | 0.810142 | 548 | (+) | RNSNNGATAANNGN | GGAAAGGTAATCAC |
|  | M00137 | V$OCT1_03 | 0.909917 | 551 | (+) | NNNRTAATNANNN | AAGGTAATCACAT |
|  | M00075 | V$GATA1_01 | 0.811945 | 553 | (-) | SNNGATNNNN | GGTAATCACA |
|  | M00076 | V$GATA2_01 | 0 | 553 | (-) | NNNGATRNNN | GGTAATCACA |
|  | M00137 | V$OCT1_03 | .827244 | 557 | (-) | NNNRTAATNANNN | ATCACATTAAAAC |
|  | M00162 | V$OCT1_06 | 0.847491 | 559 | (-) | CWNAWTKWSATRYN | CACATTAAAACATT |
|  | M00216 | V$TATA_C | 0.842187 | 560 | (+) | NCTATAAAAR | ACATTAAAAC |
|  | M00252 | V$TATA_01 | 0.848957 | 560 | (+) | STATAAAWRNNNNNN | ACATTAAAACATTCA |
|  | M00138 | V$OCT1_04 | 0.774676 | 562 | (+) | NNNNNNNWATGCAAATNNNWNNW | ATTAAAACATTCAAAGAGAATAA |
|  | M00136 | V$OCT1_02 | 0.786952 | 564 | (+) | NNGAATATKCANNNN | TAAAACATTCAAAGA |
|  | M00216 | V$TATA_C | 0.764721 | 568 | (+) | NCTATAAAAR | ACATTCAAAG |
|  | M00136 | V$OCT1_02 | 0.795615 | 574 | (-) | NNGAATATKCANNNN | AAAGAGAATAATTCT |
|  | M00223 | V$STAT_01 | 0.808419 | 591 | (+) | TTCCCRKAA | TTCACAGCA |
|  | M00162 | V$OCT1_06 | 0.840234 | 598 | (+) | CWNAWTKWSATRYN | CAGACTGTAATTAT |
|  | M00162 | V$OCT1_06 | 0.862891 | 599 | (-) | CWNAWTKWSATRYN | AGACTGTAATTATT |
|  | M00059 | V$YY1_01 | 0.803408 | 633 | (+) | NNNNNCCATNTWNNNWN | GAAGAACATTTTCTTCC |
|  | M00076 | V$GATA2_01 | 0.797474 | 4534 | (-) | NNNGATRNNN | ACTTATGGGC |
|  | M00223 | V$STAT_01 | 0.859340 | 4536 | (-) | TTCCCRKAA | TTATGGGCA |
|  | M00162 | V$OCT1_06 | 0.801172 | 6619 | (-) | CWNAWTKWSATRYN | CCCATTCCAGTACT |
|  | M00223 | V$STAT_01 | 0.812957 | 6623 | (-) | TTCCCRKAA | TTCCAGTAC |
|  | M00223 | V$STAT_01 | 0.856819 | 6635 | (+) | TTCCCRKAA | TGCCTGGAA |
|  | M00223 | V$STAT_01 | 0.832871 | 6635 | (-) | TTCCCRKAA | TGCCTGGAA |
|  | M00075 | V$GATA1_01 | 0.809970 | 6641 | (+) | SNNGATNNNN | GAAGATCCCA |
|  | M00075 | V$GATA1_01 | 0.777394 | 6641 | (-) | SNNGATNNNN | GAAGATCCCA |
|  | M00076 | V$GATA2_01 | 0.844835 | 6641 | (+) | NNNGATRNNN | GAAGATCCCA |
|  | M00076 | V$GATA2_01 | 0.848895 | 6641 | (-) | NNNGATRNNN | GAAGATCCCA |
|  | M00162 | V$OCT1_06 | 0.821484 | 6642 | (-) | CWNAWTKWSATRYN | AAGATCCCATGGAC |
|  | M00059 | V$YY1_01 | 0.793250 | 6615 | (+) | NNNNNCCATNTWNNNWN | GCACCCCATTCCAGTAC |

**Table S5.** Comparative transcription factor distribution in Mediterranean and swamp buffalo

|  | **Mediterranean Buffalo** | | | | | **Swamp Buffalo** | | | | | **P value** |
| --- | --- | --- | --- | --- | --- | --- | --- | --- | --- | --- | --- |
|  | **CSN1S1** | **CSN1S2** | **CSN2** | **CSN3** | **Total** | **CSN1S1** | **CSN1S2** | **CSN2** | **CSN3** | **Total** |  |
| **GATA** | 35 | 44 | 6 | 15 | 100 | 41 | 43 | 29 | 8 | 121 | 0.47748 |
| **TATA** | 3 | 7 | 1 | 1 | 12 | 1 | 12 | 2 | 3 | 18 | 0.375097 |
| **STAT** | 9 | 7 | 4 | 4 | 24 | 3 | 7 | 2 | 5 | 17 | 0.340442 |
| **OCT-1** | 21 | 32 | 9 | 4 | 66 | 13 | 45 | 6 | 9 | 73 | 0.729377 |
| **YY1** | 2 | 6 | 4 | 1 | 13 | 6 | 11 | 6 | 2 | 25 | 0.046205* |

[* significant value (*P<0.05*)]

**Table S6.** Casein gene family nuclear hormone receptor sites in *Bubalus bubalis* (Mediterranean breed)

| **Gene** | **Site type** | **Sequence** | **Start** | **End** | **Log**  **(Viterbi probabily)** | **log (Forward probaility)** | **log (Background state probability)** | **Forward**  **Background** |
| --- | --- | --- | --- | --- | --- | --- | --- | --- |
| **CSN1S1** | DR3 | TGAACTGACTAAACC | 3970 | 3984 | -22.4097 | -21.5208 | -23.7278 | 2.2070 |
|  | DR1 | TGAACTC  TGAAAT | 6268 | 6280 | -20.0489 | -19.2623 | -20.9351 | 1.6728 |
|  | DR1 | GGGTAAG  AGTTCA | 6781 | 6793 | -19.4465 | -19.0728 | -20.9351 | 1.8623 |
|  | DR1 | TGAACTATGAAGT | 11228 | 11240 | -20.3649 | -19.3034 | -20.9351 | 1.6317 |
|  | DR4 | TGACCTTCAGCAAACT | 13697 | 13712 | -24.8749 | -24.1178 | -25.1242 | 1.0064 |
|  | DR4 | AGGTTCTTGGAGTTCA | 14834 | 14849 | -24.7318 | -23.5682 | -25.1242 | 1.5560 |
|  | IR1 | GGGGCACTGGGCT | 14580 | 14592 | -19.9320 | -19.6185 | -20.9351 | 1.3166 |
|  | ER8 | TGTCCTTAAGGAGAAGGTCA | 5106 | 5125 | -28.2638 | -27.7854 | -30.7095 | 2.9241 |
|  | ER1 | TCAACTGAGGTAA | 5762 | 5774 | -20.7787 | -19.6077 | -20.9351 | 1.3274 |
|  | ER8 | TGAACAATTCTCTAAGGCCA | 5872 | 5891 | -30.4789 | -29.3849 | -30.7095 | 1.3246 |
|  | ER8 | TGAACTCCTCAAGCAGTGCT | 6580 | 6599 | -30.2952 | -29.4415 | -30.7095 | 1.2680 |
|  | ER4 | TGACCTGGGGAGTTCA | 7203 | 7218 | -21.5782 | -21.3327 | -25.1242 | 3.7915 |
|  | ER6 | AGAACTTTTAAAAGCTCA | 7505 | 7522 | -25.9317 | -25.5161 | -27.9168 | 2.4007 |
|  | ER1 | TGAAGTCAGTTAA | 8525 | 8537 | -20.5274 | -19.6040 | -20.9351 | 1.3311 |
|  | ER6 | GTAACTGCATTCAGTTCA | 13840 | 13857 | -26.1949 | -25.3389 | -27.9168 | 2.5779 |
|  | ER6 | TGTACTTCACTGAGTTCT | 14106 | 14123 | -26.1502 | -25.7003 | -27.9168 | 2.2165 |
|  | ER8 | TGAACTTCATCTTAAGATGA | 16551 | 16570 | -30.1020 | -28.9793 | -30.7095 | 1.7302 |
| **CSN1S2** | DR4 | GGGTCTTCCCAGTGCA | 1206 | 1221 | -24.8985 | -24.2673 | -25.1242 | 0.8569 |
|  | DR4 | GGTTCAGTGATGTTCA | 1600 | 1615 | -23.8803 | -23.5279 | -25.1242 | 1.5963 |
|  | DR2 | GGGTCACAGAGTCA | 3410 | 3423 | -22.0024 | -21.2595 | -22.3315 | 1.0720 |
|  | DR4 | TGAACTGAACTGAACT | 7672 | 7687 | -20.7567 | -20.6539 | -25.1242 | 4.4703 |
|  | IR0 | TGGCCTTGGCCC | 2449 | 2460 | -19.5341 | -18.5503 | -19.5388 | 0.9885 |
|  | IR1 | AGGTAAGAGACCT | 6777 | 6789 | -20.2790 | -19.7700 | -20.9351 | 1.1651 |
|  | IR1 | AGGCCACGGGTCT | 7163 | 7175 | -19.4035 | -19.1930 | -20.9351 | 1.7421 |
|  | IR1 | GAGCCAATGGCCT | 7188 | 7200 | -20.8086 | -20.0341 | -20.9351 | 0.9010 |
|  | IR1 | AGGTCAGTGATCA | 16580 | 16592 | -19.5858 | -19.2601 | -20.9351 | 1.6750 |
|  | ER1 | TGAACTTATTTCA | 882 | 894 | -18.3706 | -17.7778 | -20.9351 | 3.1573 |
|  | ER6 | TGACTTGCTCAAGGTTCA | 1617 | 1634 | -26.2415 | -25.8783 | -27.9168 | 2.0385 |
|  | ER8 | AGGACTTCTTTTAAAGTTCA | 2646 | 2665 | -29.9103 | -29.0062 | -30.7095 | 1.7033 |
|  | ER1 | AGACCTGGGTTCA | 3317 | 3329 | -20.3733 | -19.7057 | -20.9351 | 1.2294 |
|  | ER6 | TGAATTCAATTTAGGTAA | 4880 | 4897 | -26.4851 | -25.7995 | -27.9168 | 2.1173 |
|  | ER6 | TTAACTTTCTTTATTTAA | 4984 | 5001 | -27.1653 | -26.3011 | -27.9168 | 1.6157 |
|  | ER8 | TGAAGTTGCCACAGAGGTAA | 7829 | 7848 | -30.3711 | -29.5616 | -30.7095 | 1.1479 |
|  | ER6 | AGAACTCAAATTAATTCA | 9295 | 9312 | -27.4074 | -26.2599 | -27.9168 | 1.6569 |
|  | ER8 | TGAACTTTTGATTTGGGGCA | 13786 | 13805 | -29.8634 | -28.9736 | -30.7095 | 1.7359 |
|  | ER6 | GGATCTCCTTGCAGTCCA | 15364 | 15381 | -27.7437 | -27.0253 | -27.9168 | 0.8915 |
|  | ER6 | TGAACATCAGGAAGTTCA | 16241 | 16258 | -24.9343 | -24.2666 | -27.9168 | 3.6502 |
|  | ER4 | TGACCTTTTCAGGTCC | 16390 | 16405 | -24.4010 | -23.6089 | -25.1242 | 1.5153 |
|  | ER6 | TGTCCTTTATTGAGCTCA | 16700 | 16717 | -25.9594 | -25.2591 | -27.9168 | 2.6577 |
| **CSN2** | DR4 | TGCACTGGGACGACCC | 638 | 653 | -25.1216 | -24.4902 | -25.1242 | 0.6340 |
|  | DR8 | TGAACTCTCTATGTTAACCT | 3950 | 3969 | -30.2442 | -29.3596 | -30.7095 | 1.3499 |
|  | DR1 | TGGACTGTAACCT | 4793 | 4805 | -19.5954 | -19.2626 | -20.9351 | 1.6725 |
|  | ER8 | TGAAATGAGTCGCCAGTCCA | 584 | 603 | -29.5841 | -29.1384 | -30.7095 | 1.5711 |
| **CSN3** | DR2 | TGACCAATTCACCT | 2253 | 2266 | -21.9546 | -21.2603 | -22.3315 | 1.0712 |
|  | DR3 | TGCACTCAGTGCCCC | 4038 | 4052 | -23.5433 | -22.8926 | -23.7278 | 0.8352 |
|  | DR2 | TCAACTGCTGACCT | 4321 | 4334 | -21.2072 | -20.4851 | -22.3315 | 1.8464 |
|  | DR2 | TGAATTGATGACCT | 5680 | 5693 | -20.6860 | -20.1419 | -22.3315 | 2.1896 |
|  | DR4 | TGAATTACTATGACCT | 5832 | 5847 | -22.3609 | -22.0744 | -25.1242 | 3.0498 |
|  | DR1 | AGGTTAAAGTTTA | 5933 | 5945 | -20.2143 | -19.6165 | -20.9351 | 1.3186 |
|  | DR3 | TGAACTATTTGAGCC | 5992 | 6006 | -22.1747 | -21.9017 | -23.7278 | 1.8261 |
|  | DR1 | AGTTCATAGGTCA | 6892 | 6904 | -16.2031 | -16.1692 | -20.9351 | 4.7659 |
|  | DR2 | AGTTCATGGGGTCG | 9625 | 9638 | -21.9064 | -21.3867 | -22.3315 | 0.9448 |
|  | IR1 | AGGCCATTGACAT | 6719 | 6731 | -20.7957 | -20.0502 | -20.9351 | 0.8849 |
|  | ER8 | TTAACTTCACTTTGGGTTAA | 325 | 344 | -30.2300 | -29.2910 | -30.7095 | 1.4185 |
|  | ER6 | TGTACTTAAAATAATTCA | 2919 | 2936 | -25.9571 | -25.5521 | -27.9168 | 2.3647 |
|  | ER6 | TGTACTTTTAATATTTAA | 3087 | 3104 | -26.9806 | -26.3031 | -27.9168 | 1.6137 |
|  | ER6 | TGGACTGTAAGGAGATCC | 5363 | 5380 | -27.6543 | -26.9586 | -27.9168 | 0.9582 |
|  | ER6 | TGAAGTATAACTAGTAAA | 12231 | 12248 | -27.2707 | -26.5683 | -27.9168 | 1.3485 |
